# Characterization and organelle genome sequencing of *Pyropia* species from Myanmar

**DOI:** 10.1038/s41598-023-42262-3

**Published:** 2023-09-21

**Authors:** Myat Htoo San, Yoshio Kawamura, Kei Kimura, Eranga Pawani Witharana, Takeshi Shimogiri, San San Aye, Thu Thu Min, Cherry Aung, Moe Moe Khaing, Yukio Nagano

**Affiliations:** 1 The United Graduate School of Agricultural Sciences, Kagoshima University, Kagoshima, Japan; 2 Analytical Research Center for Experimental Sciences, Saga University, Saga, Japan; 3 Faculty of Agriculture, Saga University, Saga, Japan; 4 Graduate School of Advanced Health Science, Saga University, Saga, Japan; 5 Faculty of Agriculture, Kagoshima University, Kagoshima, Japan; 6 Mawlamyine University, Mawlamyine, Myanmar; 7 Marine Science Department, Pathein University, Pathein, Myanmar; 8 Marine Science Department, Myeik University, Myeik, Myanmar; 9 Botany Department, Dawei University, Dawei, Myanmar

**Keywords:** Comparative genomics, Conservation genomics, Genome evolution, Phylogenomics, Evolutionary biology, Biodiversity, Plant evolution, Phylogenetics

## Abstract

*Pyropia* is a genus comprising red algae of the Bangiaceae family that is commonly found in intertidal zones worldwide. However, understanding of *Pyropia* species that are prone to tropical regions remains limited despite recent breakthroughs in genomic research. Within the realm of *Pyropia s*pecies thriving in tropical regions, *P. vietnamensis* stands out as a widely recognized species. In this study, we aimed to investigate *Pyropia* species in the southwest coast of Myanmar using physiological and molecular approaches, culture-based analyses, chloroplast *rbcL* and nuclear SSU gene sequencing, and whole chloroplast and mitochondrial genome sequencing. Physiological analysis showed that the Myanmar samples were more heat-tolerant than their Japanese counterparts, including those of subtropical origin. Additionally, molecular characterization revealed that the Myanmar samples were closely related to *P. vietnamensis* from India. This study is the first to sequence the chloroplast and mitochondrial genomes of *Pyropia* species from tropical regions. A unique deletion event was observed within a ribosomal RNA gene cluster in the chloroplast genome of the studied *Pyropia* species, which is a deviation from the usual characteristics of most *Pyropia* species. This study improves current understanding of the physiological and molecular characteristics of this comparatively understudied *Pyropia* species that grows in tropical regions.

## Introduction

The red algae genus *Pyropia* from family Bangiaceae comprises members widely distributed in intertidal zones spanning subarctic to tropical regions. They are known as “nori” in Japan, “zaicai” in China, and “gim”in Korea and used in food items, such as soup, suchi, and snacks. Presently, only susabi nori [*Pyropia yezoensis* (*Neopyropia yezoensis*^1^)] and tan zicai [*Pyropia haitanensis* (*Neoporphyra haitanensis*^1^)] are commercially cultivated at scale, primarily in Japan, China, and Korea. However, numerous *Pyropia* species have been consumed and traded in these countries, prior to the onset of commercial aquaculture^2^. Increased consumption of processed *Pyropia* has estimated market value of $2 billion in 2017^3^ and reported production of two million tons in 2018^4^ alone, *Pyropia* represents one of the most valuable marine crops, globally. The health benefits, attributed to its rich vitamin, dietary fibre, protein, bioactive peptide, and lipid content, have also contributed to its growing popularity^2,5–7^.

The accurate classification of *Pyropia* sensu lato is of utmost importance, given its global distribution and economic significance. *Pyropia* is the largest and most varied genus within the bladed Bangiales and is noted for its complex taxonomy and nomenclature^8^. Historically, *Pyropia* species classification was heavily based on morphological characteristics^9^, a practice that is still prevalent today. However, relying solely on these attributes can lead to inaccuracies and misinterpretations in species biodiversity distribution^9^, complicating the accurate identification of *Pyropia* species with highly variable inter- and intra-species morphologies^9^.

Molecular techniques could be used to address these challenges. Molecular techniques with specific genetic markers have been effective in precisely identifying bladed Bangiales^10^. In earlier taxonomic classifications, the red algal genus *Porphyra* was classified under Rhodophyta, specifically within the order of Bangiales, and the name “*Porphyra*” was retained for nearly two centuries^11^. However, a substantial taxonomic revision by Sutherland et al.^8^ presented a new classification for the Bangiales taxa, recognising eight distinct genera including *Pyropia* and *Porphyra.* This reclassification was made based on molecular phylogenetic analysis, which used combined data from the nuclear SSU (nrSSU) rRNA gene and the chloroplast RuBisCO LSU (*rbcL*) gene sequences.

For a more accurate classification, Sánchez et al.^11^ added the genus *Neothemis* in 2014 based on a combined analysis of nrSSU and *rbcL* genes. This increased the number of genera within this taxonomic group to nine. Consequently, the term "bladed Bangiales" has been used to refer to all membranous species within these nine genera: *Clymene, Lysithea, Fuscifolium, Boreophyllum, Wildemania, Miuraea, Porphyra, Pyropia, and Neothemis,* offering a broad classification framework.

A recent study in 2020^11^ proposed a revision of *Pyropia* species classification based on a molecular phylogenetic analysis of nrSSU and *rbcL* gene sequences, leading to the subdivision of *Pyropia* species into six genera, including four new ones (*Calidia*, whose members primarily thrive in tropical and subtropical regions; *Neoporphyra*, primarily found in warm-temperate zones; *Neopyropia*, mostly found in cold-temperate regions; and *Uedaea*), one resurrected genus (*Porphyrella*), and one redefined genus (*Pyropia*). However, the evolutionary process of *Pyropia* may be more convoluted than what can be derived from the analysis of just two genes, indicating potential complexity in the genetic relationships within the newly proposed classification system.

Additionally, a proposal has been made to restore the genus *Pyropia* to its original definition before its redefinition^12^. More recently, a report^13^ proposed a new genus name “*Phycocalidia*” to replace the red algal genus “*Calidia* Yang, L.-E and Brodie, J.” and recommended transferring species previously assigned to *Calidia* to their newly proposed genus, “*Phycocalidia*”. However, in our study, we primarily used the original *Pyropia* name^8^, adding the newly proposed name in parentheses where necessary. The basionym, homotypic synonyms, and current taxonomic classification of the species studied are listed in Supplementary Table S1.

In addition to well-documented genes, the organelle genomes, including chloroplast and mitochondrial genomes, contain valuable genetic data due to their large DNA regions^14^. In particular, chloroplast genomes has been widely used in plant science for phylogenetics, phylogeography, and population genetics, thanks to its distinctive characteristics such as uniparental inheritance and conserved genome structure. The advent of high-throughput sequencers has revolutionized genome sequencing approaches, making them more affordable and accessible. Moreover, organelle genome databases provide valuable insights into the phylogenetic relationships and molecular evolution of various species. For example, mitochondrial and chloroplast genome data have been crucial in reestablishing the phylogenetic relationships among red algae^15–17^.

Seaweeds are regarded as reliable natural resources in Myanmar^18^. In 1975, Thein and Myint^19^ first reported the occurrence of *Porphyra crispata* Kjellman (*Phycocalidia acanthophora*^13^) in southern Myanmar. Later in 1999, Sein^20^ identified *P. crispata* in her Master's thesis; in 2003, she reclassified *Porphyra crispata* as *Pyropia suborbiculata*^21^ (*Phycocalidia suborbiculata*^13^). Both studies^20,21^ performed extensive investigations of the morphologies and performed laboratory cultures under various environmental conditions to confirm its identity. However, no *Pyropia* species from Myanmar has yet been subjected to molecular characterisation. Therefore, undertaking molecular analyses of *Pyropia* species, especially those from tropical regions, would contribute to a better understanding of the evolutionary dynamics of the *Pyropia* genus.

Among the different types of *Pyropia* species grown in tropical regions, *P. vietnamensis* (*Phycocalidia vietnamensis*^13^) has a wide distribution in the northern Indo-Pacific. *P. vietnamensis* was first reported in Vietnam^22^, as a species that grows in the upper intertidal zone. The species has monostromatic thalli with undulate margins and well-developed spinulate processes. This species has also been reported in India^23^ and Thailand^24^. Despite being well-known as a species native to tropical areas, a previous study indicated its occurrence in the southwestern Atlantic, suggesting that it might have been introduced from the tropical Indo-Pacific regions^25^. It remains unknown whether the *Pyropia* species found in Myanmar are related to *P. vietnamensis.*

The current study aims to identify and characterise *Pyropia* species from Myanmar by documenting their chloroplast and mitochondrial genomic sequences. Additionally, we explore the phylogenetic relationships of *Pyropia* species using a range of datasets, including combined data of nrSSU rRNA gene and *rbcL* genes, organellar genome sequences, and protein sequences. By integrating these diverse datasets, we aim to provide a comprehensive understanding of the evolutionary relationships within the genus *Pyropia* and provide insights into the evolutionary history of the species from Myanmar.

## Results

### Sample collection from Myanmar

Nineteen samples were collected from the southwestern coast of Myanmar (Supplementary Fig. S1). The sample collection site was probably the same as, or in close proximity to, the location noted in previous reports^20,21^. Sixteen samples were dried for DNA analysis, and the remaining three were used for culture experiments and DNA analysis. Samples used in the culture experiment exhibited differences in morphology and were referred to as strains Myanmar A, B and C (Supplementary Fig. S2). All three strains had undulating blade-shaped thalli. Thalli of strain Myanmar A and C were linear lanceolate in shape, whereas those of strain Myanmar B were orbicular to ovate. In contrast to strain Myanmar A that grew as individual filaments, thalli of strain Myanmar C grew in clusters (Supplementary Fig. S2).

### Molecular characterization based on combined dataset of nrSSU and *rbcL* sequences

Polymerase chain reaction (PCR) of these 19 samples yielded 1381 bp DNA fragments for *rbcL* gene. DNA sequencing of the PCR products revealed that the sequences of all 19 samples were identical. The phylogenetic tree based on *rbcL* sequences showed that the collected specimens are closely related to *Pyropia vietnamensis* (*Phycocalidia vietnamensis*^13^) from India and *Pyropia* sp. from Piaui State, Brazil^22^, which has already been classified as *Phycocalidia vietnamensis*^13^ (Supplementary Fig. S3). The origin of related *Pyropia* species used in analysis are summarised in Supplementary Table S2.

**Figure 1:**
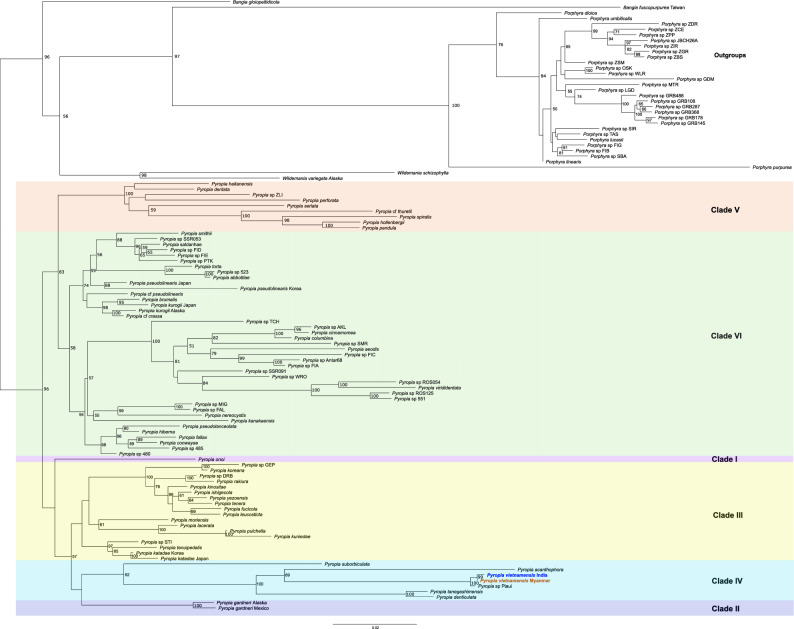
Maximum likelihood phylogram of 111 Bangiales taxa computed from the concatenated nuclear SSU ribosomal RNA (nrSSU) and RuBisCO LSU (*rbcL*) data from Myanmar specimens, and the dataset described by Sutherland et al.^8^ (TreeBASE ID: S11223) using RaxML. The numbers at the nodes represent bootstrap values (% over 500 replicates). The scale bar denotes the number of substitutions per site. Samples from Myanmar are highlighted in orange. The clade names used in the figure are those described by Yang et al.^1^. Bootstrap values less than 50% were not shown. A total of 3260 nucleotide positions (804 parsimony informative sites) were used in this analysis. The alignment data is available in fasta format as Supplementary Dataset 3, and ML tree is available in newick format as Supplementary Dataset 4.

Combined *rbcL* and nrSSU data are frequently used in phylogenetics in Bangiales, although discrepancies between nuclear- and chloroplast-based phylogenetic trees have been reported^26,27^, especially in higher plants. Therefore, we assembled nrSSU sequences for strains Myanmar A, B, and C using data high-throughput DNA sequencing (Supplementary Table S3). Phylogenetic analysis based on combined dataset of nrSSU and *rbcL* sequence data again revealed that these strains are closely related to *Pyropia vietnamensis* (Fig. 1 and Supplementary Fig. S4). Based on the results of these molecular analyses, we tentatively identified the Myanmar samples as *Pyropia vietnamensis*.

### Culture-based physiological analysis revealed that the Myanmar *Pyropia* species are adapted to high temperatures

**Figure 2:**
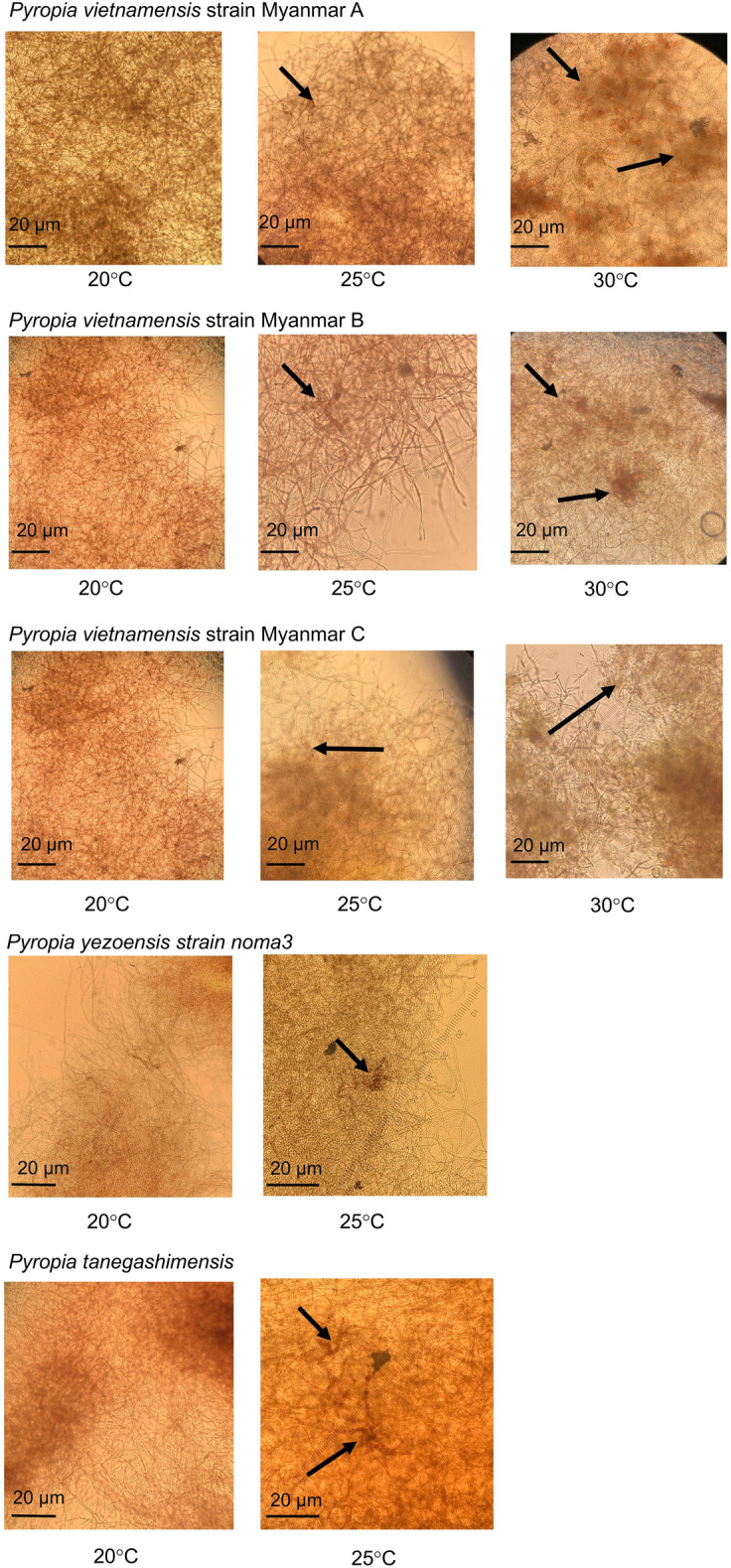
Growth of conchocelis filaments of *Pyropia vietnamensis* strain Myanmar A, B, C, *Pyropia yezoensis* strain noma3 gou, and *Pyropia tanegashimensis* at different temperatures (20, 25 and 30 °C). Formation of conchosporangia is indicated with arrows.

Given the sea temperature during the sample collection (approximately 30 °C), the previously characterised optimum growth temperature for the conchocelis (one of vegetative stages) of *Pyropia* species in Myanmar (20–30 °C) by Sein et al.^20,21^, and the growth temperature range for the comparative sample, conchocelis of *Pyropia yezoensis* (15–25 °C), we decided to conduct comparative growth experiments in the 20–30 °C range. We cultured *Pyropia yezoensis* strain noma3 gou that is typically grown in cold-temperate regions^28^ of Japan, *Pyropia tanegashimensis* that is grown in subtropical^29^ regions of Japan, and *Pyropia vietnamensis*^12^ that is grown in tropical regions from Myanmar (three strains) to investigate their physiological differences. During the conchocelis stage, all three *Pyropia* species showed good growth in the static culture at 20 °C and 25 °C. However, only the Myanmar samples exhibited growth at 30 °C; no growth was observed for the two Japanese species (Fig. 2, Supplementary Figs. S5 and S6).

Analysis of the conchosporangia stage (a vegetative stage) showed that none of the Myanmar samples or *P. tanegashimensis* exhibited any conchosporangia growth, whereas *P. yezoensis* exhibited conchosporangia growth at 20 °C (Fig. 2). However, at 25 °C and 30 °C, all Myanmar *Pyropia* species exhibited conchosporangia growth, with a better formation rate at 30 °C (Supplementary Fig. S7). Both *P. yezoensis* and *P. tanegashimensis* exhibited conchosporangia growth at 25 °C but not at 30 °C (Fig. 2, Supplementary Fig. S7).

We performed an aeration culture of Myanmar samples using conchosporangia and examined its gametophytic life cycle (reproductive stage). The samples were cultured at 20, 25, and 28 °C and the release of conchospores from conchosporangia was observed. The Myanmar samples demonstrated the highest conchospore release rate at 28 °C (Supplementary Fig. S8), although the differences among the three Myanmar strains were large. Similarly, measurement of the leaf area (leafy gametophyte) of the *P. vietnamensis* strain Myanmar A confirmed that 28 °C is the optimum temperature for conchosporangia growth (Supplementary Fig. S9).

Next, taking into account previous reports^20,21^, we investigated how different conditions, namely, varying salinities (15 and 30 psu) and temperatures (26, 28, and 30 °C) impacted the growth of the natural spore germlings (a gametophytic stage) of *P. vietnamensis* from Myanmar. During the first week, growth was observed under almost all the tested conditions with *P. vietnamensis* strain Myanmar A being the only exception at 15 psu and 26 °C. However, by the end of the second week, optimal growth was only observed at 30 °C and 15 psu for all samples except in *P. vietnamensis* strain Myanmar A, which showed growth at 30 °C and 30 psu.

**Figure 3:**
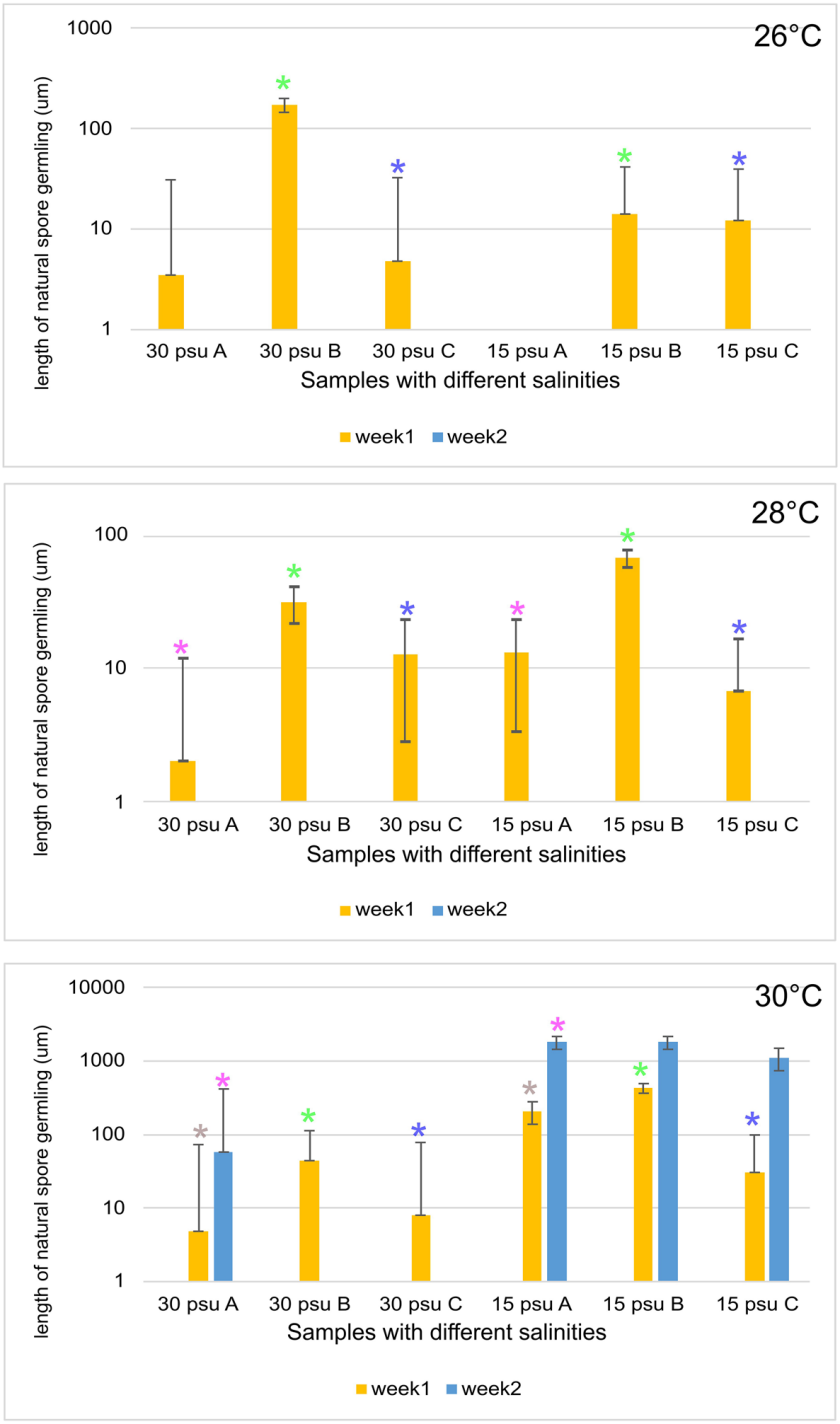
Effects of salinity and temperature (26, 28, and 30 °C) on the growth of natural spore germlings of *Pyropia vietnamensis* strain Myanmar A, B and C. No spore germling were observed at 26 °C in *P. vietnamensis* strain Myanmar A at 15 psu. Error bars represent standard deviation of triplicate cultures. Statistical significance was calculated by t-test and asterisk above the histograms indicates significant difference level: *P < 0.05. The data being compared are represented by specific colored asterisk for clarification.

We further investigated the Myanmar samples to determine whether they exhibited monoecious or dioecious characteristics. *Pyropia* species exhibited a heteromorphic life cycle with alternating leafy gametophyte and filamentous sporophyte (known as the conchocelis stage) stages^19^. During sexual reproduction, vegetative cells produce carpogonia and colourless spermatangia either on the same (monoecious) or different (dioecious) thalli. We identified both the carpogonium and spermatangium in the same thallus, indicating the monoecious nature of the species (Supplementary Fig. S10) (Fig. 3).

### Chloroplast genomes of *Pyropia* species from Myanmar lack rRNA repeats, which is characteristic of most *Pyropia* species

**Figure 4:**
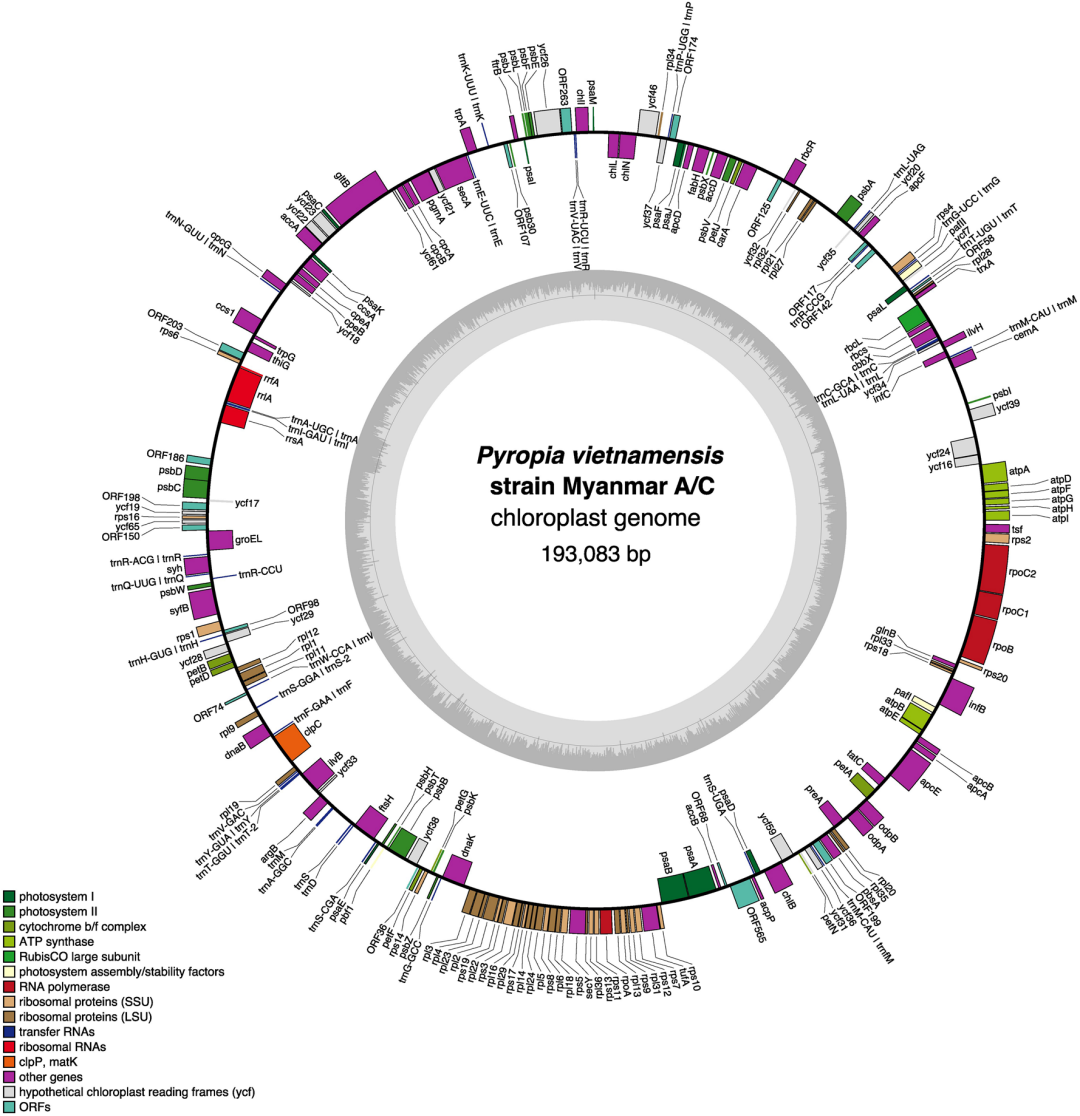
Physical map of the chloroplast genome of *Pyropia vietnamensis* strain Myanmar A/C created using OrganellarGenomeDraw (OGDRAW; ogdraw.mpimp-golm.mpg.de). Genes are coloured according to their function. Anticlockwise transcribed genes are on the outer side and clockwise transcribed genes are on the inner side of the circle. Dashed area in the inner circle indicates the guanine–cytosine (GC) content of the organelle genomes.

We assembled the chloroplast genome sequences using data from high-throughput DNA sequencing (Supplementary Table S3). We validated the accuracy of the de novo assembly by mapping the short reads back to this assembled chloroplast genome sequence, which confirmed the robustness of our assembly results (Supplementary Fig. S11). Mapping results revealed the absence of heteroplasmy. Chloroplast genome sequences of *P. vietnamensis* strain Myanmar A, B and C were 193,083, 193,090, and 193,083 bp, respectively (Fig. 4, Supplementary Fig. S12) and showed a characteristic circular structure with a guanine–cytosine (GC) content of 32.06%. Comparative genome analysis of the three strains revealed that the chloroplast genome sequences of strains Myanmar A and C were identical. However, comparing strain Myanmar B with strain Myanmar A/C, we identified seven gaps and 11 mismatches. The order of genes in the chloroplast genomes of all *P. vietnamensis* strains from Myanmar remained invariant. Annotation results revealed that only one region carried the ribosomal RNA (rRNA) genes in all Myanmar samples, although most other *Pyropia* species carry direct repeats of rRNA genes^30–32^. The complete absence of repeats in the Myanmar samples was confirmed via dot plot analysis (Supplementary Fig. S13) and through the lack of doubling of the depth in the rRNA gene region in mapping (Supplementary Fig. S11).

### Loss of rRNA repeats in the Myanmar *Pyropia* species is different from the pattern observed in Korean *Pyropia dentata*

**Figure 5:**
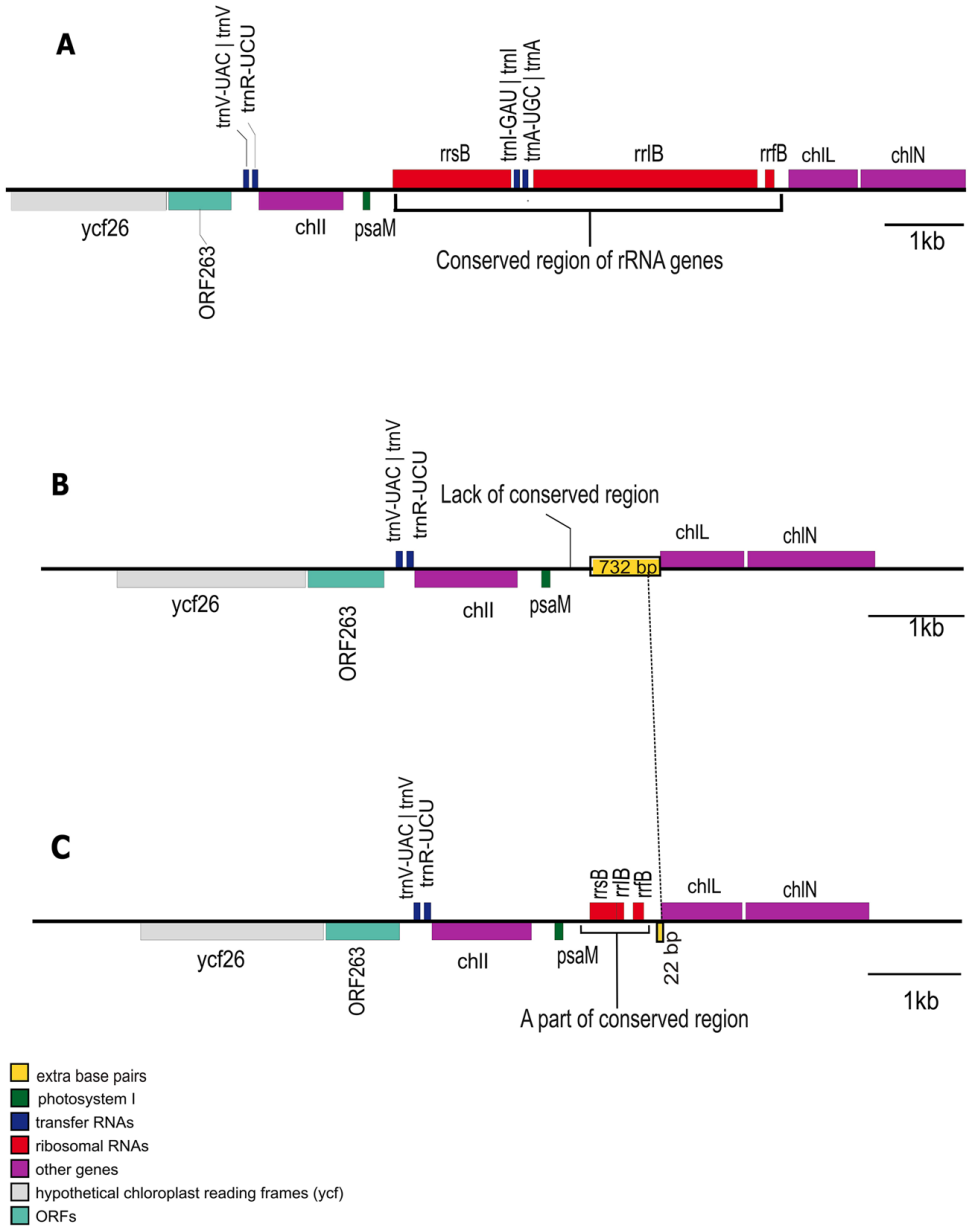
Comparisons of the rRNA repeat regions of (A) Japanese seaweed (*P. yezoensis*), (B) Myanmar seaweed (*P. vietnamensis*), and (C) Korean seaweed (*P. dentata*).

Analysis of the rRNA regions of the chloroplast genomes of various *Pyropia* species revealed that the loss of rRNA repeats occurred not just in *Pyropia* from Myanmar but also in *Pyropia dentata* (*Neoporphyra dentata*) from Korea^33^, which usually grows in warm-temperate regions in East Asia. To further investigate this phenomenon, we conducted a comparative study of *P. vietnamensis* strain Myanmar A/C, *P. yezoensis* and *P. dentata* (Fig. 5). (The *P.yezoensis* species analysed in this comparative study was classified as originating from China^30^. It is important to note the fact that the collection site was a nori farm in China, as outlined in the study^30^. However, another study^34^, which delved into the diversity of *Pyropia* species, inferred that the cultivated Chinese *Pyropia* are genetically similar to Japanese ones but not to Chinese wild ones from Shandong Province, China. Further research is necessary to fully understand the origin of the species.) Comparison of *P. yezoensis* and *P. vietnamensis* strain Myanmar A/C revealed two key differences: 1) The *P. vietnamensis* strain Myanmar A/C lacked the conserved unit of the rRNA genes that are present at two locations in *P. yezoensis*; 2) The *P. vietnamensis* strain Myanmar A/C had a 732 bp DNA sequence insertion that was not present in *P. yezoensis*. In *P. dentata*, the conserved unit of the rRNA genes was not completely deleted, and the head and tail regions of this unit remained. In particular, *rrfB* (encoding 5S rRNA B) remained completely intact, and parts of *rrlB* (encoding 23S rRNA B) and *rrsB* (encoding 16S rRNA B) remained. Further information regarding the rRNA cluster for all the species examined in the subsequent phylogenetic analysis is summarised in Supplementary Table S4. Moreover, a 22 bp region in *P. dentata* exhibited a similarity to the unique 732 bp DNA sequence found in the Myanmar sample. No sequence demonstrating similarity to the full 732 bp from the Myanmar sample was identified in the existing database.

### Mitochondrial genome sequencing and analysis

**Figure 6:**
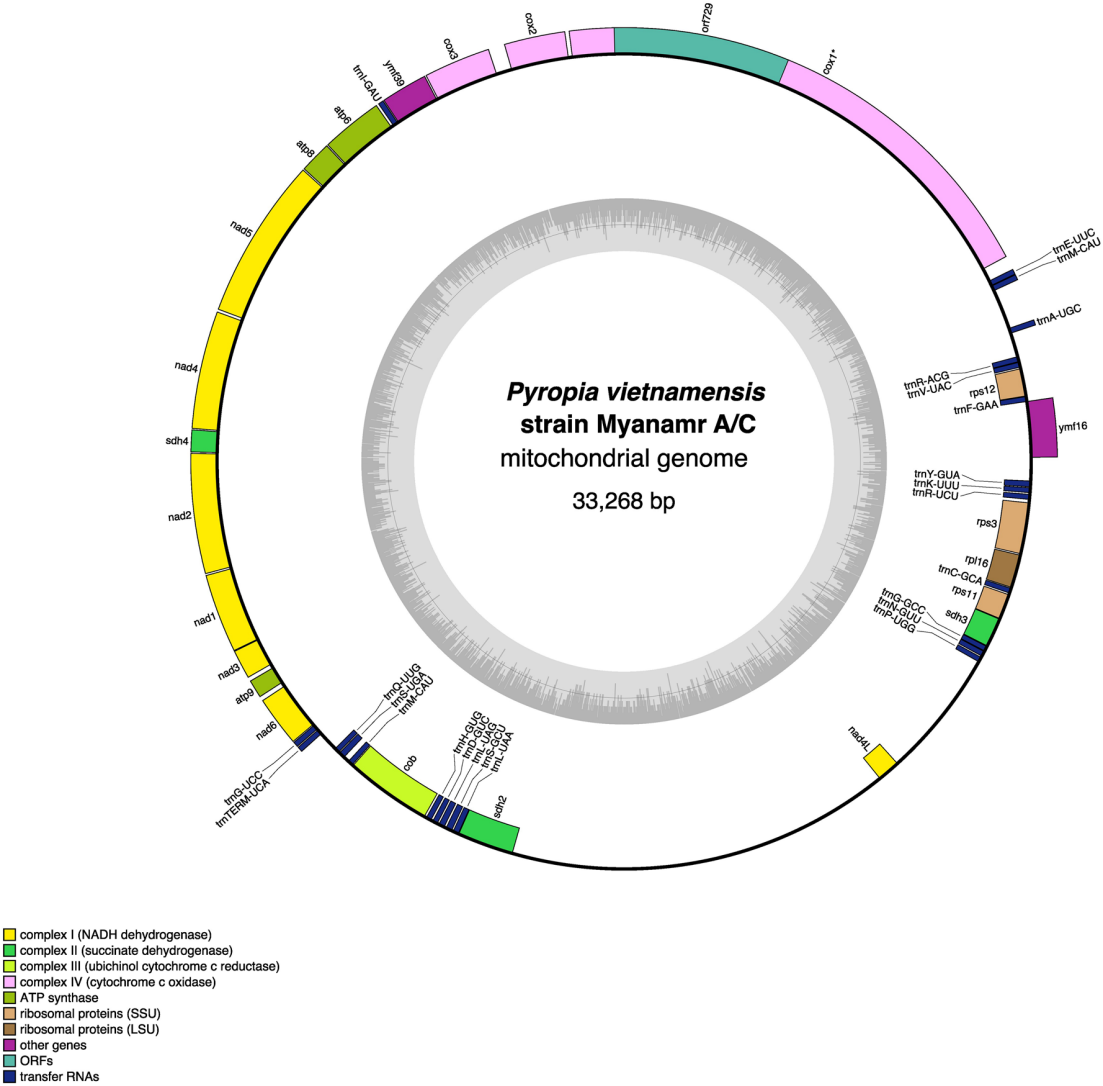
Physical map of the mitochondrial genome of Myanmar seaweed created using OGDRAW (ogdraw.mpimp-golm.mpg.de). Genes are coloured according to their function. Anticlockwise transcribed genes are on the outer side and clockwise transcribed genes are on the inner side of the circle. Dashed area in the inner circle indicates the guanine–cytosine (GC) content of the organelle genomes.

We assembled the mitochondrial genome sequences of the Myanmar samples. Mapping of short reads to the assembled sequence revealed the presence of uncertain deep regions with multiple DNA sequences (Supplementary Fig. S14). We sequenced this region via Sanger sequencing and successfully removed the presumptive nuclear DNA sequence that was incorrectly mapped. Therefore, heteroplasmy was not observed in this region. The mitochondrial genome sequences of *P. vietnamensis* strain Myanmar A, B and C were 33,268, 33,267, and 33,268 bp long, respectively, exhibiting a typical circular structure (Fig. 6 and Supplementary Fig. S15) and GC content of 29.51%. A comparative analysis of the genomes of the three strains revealed that the mitochondrial genome sequences of *P. vietnamensis* strain Myanmar A and C were identical. However, *P. vietnamensis* strain Myanmar B displayed three gaps and six mismatches compared with *P. vietnamensis* strain Myanmar A/C.

### Phylogenetic analysis of organelle genomes

To establish the systematic position of the *P. vietnamensis* strain Myanmar A/C and B, we used the Maximum Likelihood (ML) and Bayesian inference (BI) phylogenetic method to construct phylogenetic trees for the chloroplast (Supplementary Figs. S16 and S18) and mitochondrial (Supplementary Figs. S17 and S19) genomes using the genomes of members of the *Bangiaceae* family available on the DDBJ/EBI/GenBank nucleotide sequence databases (Supplementary Tables S5 and 6). In this analysis, we used only highly conserved DNA sequences extracted using Homblocks^35^ and trimal^36^. The topology of the BI tree and the ML tree were consistent with each other. However, a discrepancy was noticed in the topology between the phylogenetic trees of the chloroplast and mitochondrial genomes. In the chloroplast phylogenetic tree, clade IV (the group containing the newly proposed genus *Phycocalidia*), to which the *P. vietnamensis* strain Myanmar A/C and B belonged, was an outgroup of clades III, VI, and V (the groups containing the newly proposed genera *Neopyropia*, *Pyropia*, and *Neoporphyra*, respectively). Furthermore, within the group containing clades III, V, and VI, clade III was an outgroup of clades V and VI; clade V was sister to clade VI (Supplementary Figs. 16 and 18). However, in the mitochondrial phylogenetic tree, clade V was sister to clade VI and clade III was sister to clade IV, which the *Pyropia* species from Myanmar belonged to. Furthermore, a large clade containing clades V and VI was sister to a large clade containing clades III and IV (Supplementary Figs. 17 and 19).

**Figure 7:**
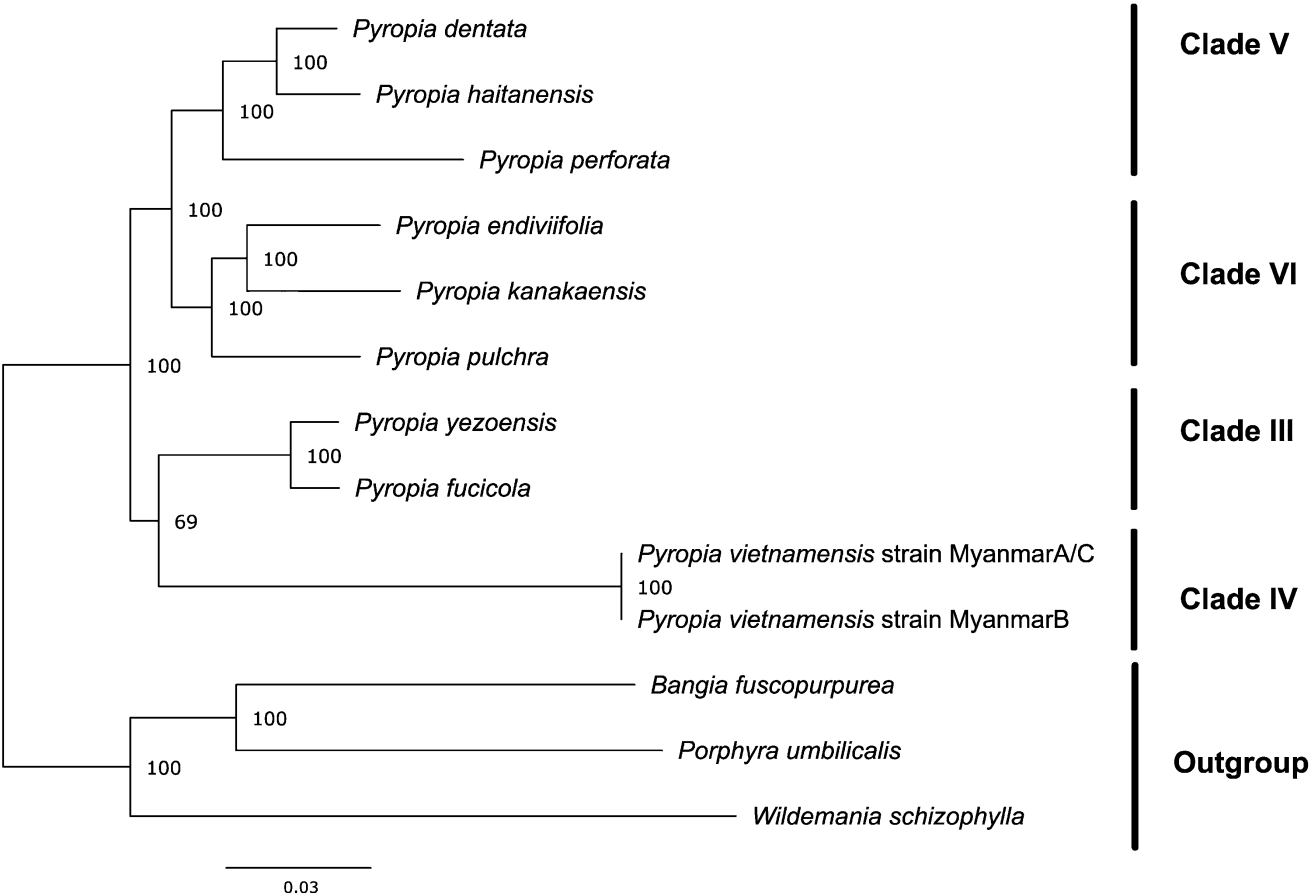
Maximum likelihood (ML) tree of chloroplast genomes of *Pyropia* species. Protein sequences were extracted from the chloroplast genome dataset of Myanmar seaweed and from available sequence data in the database. Orthologous groups were extracted by using Orthofinder. Bootstrap support values are presented at each node. A total of 28,466 amino acid positions (4072 parsimony informative sites) were used in this analysis. The origin of the samples used in this analysis are described in Supplementary Table 5. The alignment data are available in fasta format as Supplementary Dataset 13, and ML tree is available in Newick format as Supplementary Dataset 14.

**Figure 8:**
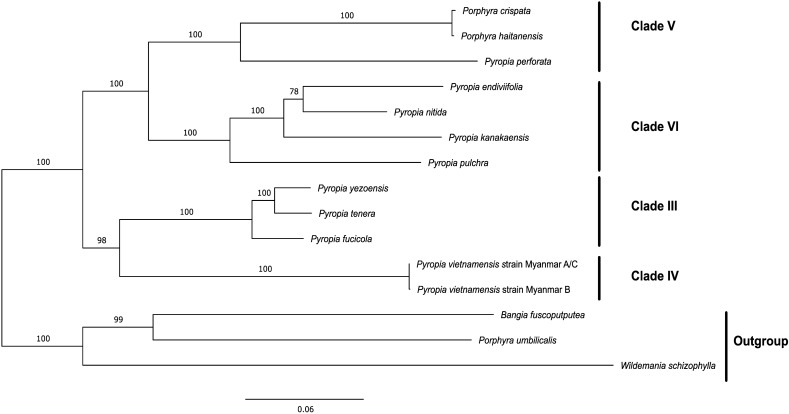
Maximum likelihood (ML) tree of mitochondria genomes of *Pyropia* species. Protein sequences were extracted from the mitochondria genome dataset of Myanmar seaweed and from available sequence data in the database. Orthologous groups were extracted by using Orthofinder. Bootstrap support values are presented at each node. A total of 5455 amino acid positions (1514 parsimony informative sites) were used in this analysis. The origin of the samples used in this analysis is described in Supplementary Table 6. The alignment data are available in fasta format as Supplementary Dataset 15, and ML tree is available in Newick format as Supplementary Dataset 16.

The ML tree constructed based on the protein sequences from the chloroplast genome suggested that the clade comprising *P. vietnamensis* strain Myanmar A/C and *P. vietnamensis* strain Myanmar B was the sister group to clade III representing genus *Neopyropia* (Fig. 7). Clade V and clade VI were found to be sister groups. Additionally, a larger clade containing clades IV and III was the sister group to another large clade containing clades V and VI. Hence, inconsistencies were observed in the phylogenetic tree constructed from protein and nucleotide sequences derived from the chloroplast genome. In contrast, the maximum likelihood (ML) analysis results using protein sequences from mitochondrial genomes revealed a consistent evolutionary pattern with the results obtained from nucleotide data (Fig. 8). Notably, the regions of DNA sequences used in the phylogenetic analysis and the regions of protein sequences used in the phylogenetic analysis differ because highly conserved regions were extracted independently for both analyses.

## Discussion

The combined molecular characterization of nrSSU and *rbcL* sequences (Fig. 1) suggests a close relationship between *Pyropia* sp. strains from Myanmar and *P. vietnamensis (Phycocalidia vietnamensis*^13^*)* from India (Fig. 1 and Supplementary Fig. S2). However, the current identification of *P. vietnamensis* has not been sufficiently validated. Initially, the species was named *P. vietnamensis* based on morphological characteristics^22^ with the taxon sampling site reported as Vietnam. Subsequently, Sutherland et al. revised the classification to *P. vietnamensis* and analysed the *rbcL* and nrSSU sequences of a sample collected from Thangeseri (Kerala), India. Another study^23^ analysed the *cox1* sequence of *P. vietnamensis* but the collection site was the Konkan region of India. Nevertheless, the connections between these two publications^22,23^ are not readily apparent. We collected the *P. vietnamensis* seaweed samples from the Bay of Bengal, which is a part of the Indian Ocean. However, the taxonomy of *Pyropia* species in the Indian Ocean has not yet been fully explored. Furthermore, data on the *rbcL* sequence, which is widely acknowledged as one of the best barcode genes, are currently lacking for specimens collected from Vietnam. Therefore, further identification is urgently warranted, and the identification of *P. vietnamensis* itself may require revision. For the time being, however, we have tentatively labelled our samples as “*Pyropia vietnamensis*”.

Our study aimed to evaluate the heat tolerance properties of Myanmar seaweed through a comparative physiological analysis. The results demonstrated that these seaweeds possess the ability to survive under high temperatures, specifically at 30 °C, indicating their inherent heat tolerance (Fig. 2 and Supplementary Fig. S5 and 6). High-temperature stress is a significant concern for seaweeds worldwide, including those from the genus *Pyropia*. Recent observations have shown a drastic decline in *Pyropia* growth due to high-temperature stress^37^, prompting research endeavours focused on enhancing heat tolerance in these species. Some strategies being explored include the screening of heat-resistant mutants in *Pyropia haitanensis*^38^ and the induction of mutations in *Pyropia tenera* using gamma irradiation^39^. The analysis of seaweed resources from tropical regions holds great potential because these organisms have naturally adapted to thrive in high-temperature environments, distinguishing them from their counterparts in colder regions. Consequently, investigating the mechanisms underlying high heat tolerance of Myanmar seaweeds is of utmost importance in advancing our understanding of seaweed biology and facilitating future research efforts in this field.

However, the absence of conchosporangia growth in Myanmar seaweed at 20 °C (Fig. 2) revealed its intolerance to relatively low water temperatures not often observed in the tropical regions. The physiological analysis showed no conchocelis maturation in Myanmar seaweed at 20 °C (Fig. 2), although conchocelis growth was observed at the same temperature in the sporophyte stage (Supplementary Fig. S5). Hence, our findings affirm not only the heat tolerance characteristics of Myanmar seaweed but also its inherent intolerance to relatively low temperatures, a prevalent trait among seaweeds thriving in tropical regions.

Our analysis of the growth of natural spore germlings under different temperatures and salinities confirms the results of a previous study^20,21^. Temperature and salinity are the two main environmental factors affecting vegetative and reproductive growth and the liberation and germination of spores in seaweed^40^. However, the optimal conditions for these processes can vary depending on the natural habitat of the species. For example, in Japanese seaweed, the suitable salinity range for the formation of natural spore germling is 30–40 psu, whereas that for the Myanmar seaweed at the collection site was 10–20 psu during the natural spore germling season^18^. Our study determined that the best conditions for Myanmar seaweed spore release were 30 °C temperature and 15 psu salinity (Fig. 3), which was consistent with the findings of Sein^21^.

The fact that the collection sites were on the same coast suggests that the red algae investigated in previous studies^20,21^ and those in our current study are the same species. Molecular phylogenetic analysis using *rbcL* and nrSSU sequences suggested a slight discrepancy in the species identification in previous studies involving the Myanmar specimen. Thein and Myint identified this red alga as *Porphyra crispata*^19^ (*Phycocalidia acanthophora*^13^). In contrast, Sein identified it as *Porphyra suborbiculata*^21^ (*Phycocalidia vietnamensis*^13^). However, our study revealed that the red algae growing on the coast of Myanmar are similar to *Pyropia vietnamensis* [*Phycocalidia vietnamensis*^13^]. Therefore, we hypothesised that previous studies may have erred by relying solely on morphological characteristics. Notably, *P. crispata*, *P. suborbiculata*, and *P. vietnamensis* belong to clade IV (Fig. 1, Supplementary Fig. S2), which contains the newly proposed genus *Phycolacilia*, but occupy distinctly different positions within the clade. Based solely on morphological characterisation, Sein^21^ successfully inferred that this red alga belongs to this group.

Among red algae, the first organelle genome sequencing was performed for *Porphyra purpurea*^32^*.* A subsequent study has identified organelle genomes in other members of the family *Bangiaceae*, including in *P. yezoensis*, *Pyropia haitanenensis*, and *Pyropia tenera*^41–43^. The chloroplast genome of the members of *Bangiaceae* is characterised by the presence of two direct repeats carrying rRNA genes^31,32^, although the two sequences of the conserved unit of the rRNA genes are marginally different. However, one region carrying the rRNA gene was found to be missing in the Myanmar samples (Fig. 4; Supplementary Figs. S12 and 13). Similar characteristics were observed in *P. dentata* from Korea^33^. Interestingly, *P. dentata* collected from Japan^34^ does not show the characteristics of lacking one repeat. (Given that the two sequences of the conserved unit of the rRNA genes of *P. dentata* (*Neoporphyra dentata*) in Japan have not yet been determined, this Japanese seaweed was not included in the phylogenetic analysis of the current study). Phylogenetic analysis suggested that the Myanmar and Korean seaweeds were not closely related (Fig. 7). Therefore, the loss of one direct repeat in the chloroplast genome may have occurred independently.

That the loss of one direct repeat occurred independently is plausible. However, the hypothesis that the loss happened simultaneously and that the loss was detected in only two types of seaweed because of mechanisms such as incomplete lineage sorting is equally possible. If this loss occurred simultaneously, the location of the deletion detected in the two types of seaweed might be the same. However, as shown in Fig. 5, the locations of the deletions differed. This observation strongly supports the hypothesis that one of the direct repeats in the chloroplast genome was lost independently.

To the best of our knowledge, this study is the first to analyse the organelle genome of clade IV (*Phycocalidia*) within *Pyropia* sensu lato. As previously mentioned, *Pyropia* sensu lato has been divided into six clades^1^. Our samples belong to this clade including both *Pyropia tanegashimensis* and *Pyropia vietnamensis*. Prior to this study, no organelle genome sequence had been established for this clade. The results of our analysis suggest phylogenetic conflicts among the clades. Our comparative phylogenetic analysis, utilizing protein and nucleotide sequences, revealed discrepancies (Fig. 7, Supplementary Figs. S16, and S18). The nucleotide-based phylogenetic tree positions clade IV, consisting of *P. vietnamensis* strains Myanmar A/C and B, as the outgroup among the clades. In contrast, the protein-based phylogenetic analysis indicates that clade IV is related to clade III. This conflicting pattern between the two types of sequences may arise from rapid or multiple substitutions in the chloroplast nucleotide sequences and/or limited taxon sampling. Moreover, protein-based phylogenetic analyses are generally considered more reliable than nucleotide-based analyses due to their lower susceptibility to nucleotide compositional bias and slower functional changes during evolution^44^. Therefore, we regard this discrepancy as an artifact and rely solely on the phylogenetic analysis based on protein data. The fact that the mitochondrial phylogenetic tree based on protein sequences and DNA sequences matches the chloroplast phylogenetic tree based on protein sequences supports this speculation.

We delved into the genealogical history of red algal species by comparing various phylogenetic trees (Supplementary Fig. S20). We referenced cladograms from prior phylogenetic studies by Sutherland et al.^8^ and Zuccarello et al.^12^ (Supplementary Fig. S20A), as well as Yang et al.^1^ (Supplementary Fig. S20B), both of which were built upon concatenated short sequences of *rbcL* and ribosomal RNA. Furthermore, we incorporated cladograms generated from our own research, based on concatenated sequences of *rbcL* and nrSSU (Supplementary Fig. S20C), as well as protein sequences extracted from chloroplast and mitochondrial genomes (Supplementary Fig. S20D).

When scrutinizing three phylogenetic trees predicated on short sequence data (Supplementary Fig. S20A–C), we observed topological discrepancies, despite all three focusing on identical sequence data for *rbcL* and ribosomal RNA. For instance, the Maximum Likelihood (ML)-based Supplementary Fig. S20A exhibits clades III, IV, and II forming a monophyletic group, with clade III serving as the outgroup among them. Simultaneously, clades V and VI form another monophyletic group, and these two monophyletic groups emerge as sister taxa. In contrast, the Bayesian Inference (BI)-based Supplementary Fig. S20B posits that clade III diverged early, followed by clade IV and a monophyletic group comprising clades VI and V. Our current study (Supplementary Fig. S20C) lends support to the phylogeny presented in Supplementary Fig. S20A, albeit with some exceptions such as the ambiguous position of clade I, which had low bootstrap support (bootstrap value of less than 50).

These conflicting topological outcomes could arise from a multitude of variables, including choice of phylogenetic methods, software, model selection, and data availability. Different software tools employ unique algorithms and model frameworks, leading to divergent results^45^. Moreover, the type of data used can significantly affect the topology. Short sequences may lack the requisite information for precisely elucidating complex evolutionary relationships and may also pose challenges in alignment, particularly in highly variable regions^46^. Taxon sampling can also influence the phylogenetic tree's topology. A greater number of taxa could enhance the accuracy of phylogenetic inference by increasing the likelihood of detecting non-random patterns^47^. A recent study^12^ revisited the data presented by Yang et al.^1^, integrating additional insights obtained through alternative phylogenetic methodologies, thereby refining the existing *Pyropia* phylogeny.

Given these complexities, relying solely on short sequence data for phylogenetic tree construction is precarious. It underscores the importance of careful gene and method selection, as well as meticulous analysis of support metrics such as bootstrap values. As a response, we employed longer, more reliable sequences for our phylogenetic analysis, which focused on protein genome sequences from mitochondria and chloroplasts (Supplementary Fig. S20D). Our results substantiate the existence of two monophyletic clusters, each supported by robust evidence, thus lending greater credence to the phylogenies presented in Supplementary Fig. S20A and C, but not to Supplementary Fig. S20B.

To sum up, while all phylogenetic trees confirm the monophyletic clustering of clade VI and V, the positions of clades I, II, III, and IV remain ambiguous. Previous studies used short DNA sequences, but our utilization of extensive DNA sequences from organelle genomes enhances both the reliability and precision of our findings. Nonetheless, the paucity of genomic data for taxa within clade I and II continues to pose a challenge for comprehensive confirmation.

In conclusion, this study presents the chloroplast and mitochondrial genomes of *Pyropia vietnamensis*, a species from the tropical region of Myanmar. We have also shed light on the evolutionary history of *Pyropia* species through genomic analysis. However, due to the uncertainty associated with short-sequence data, as well as the limited availability of genomic data based on long-sequence data, establishing an accurate phylogeny of *Pyropia* species remains a challenge. Therefore, further research is essential, particularly involving samples from unexplored oceanic regions such as the Indian Ocean. Additionally, obtaining comprehensive information about the organellar genomes of *Pyropia* species is of significant importance. These efforts are poised to contribute substantially to a deeper understanding of the evolutionary dynamics and phylogenetic relationships within the *Pyropia* genus.

## Methods

### Sample collection and preparation

*Pyropia* samples were collected from a location near Maw Tin Point (North latitude 16.03428, East longitude 94.20035; Supplementary Fig. S1) on the southwest coast of Myanmar during the rainy season (August 2019). All specimens were handpicked, and the collected samples were stored in two conditions: semi-dry (− 80 °C) and/or fresh (15 °C).

### Physiological analysis based on culture

Three Myanmar individuals (*P. vietnamensis* strains MyanmarA, B, and C) were used in this study. *P. tanegashimensis* used in this study was obtained from Dr. Masahiro Notoya of Tokyo University of Marine Science and Technology. *P. yezoensis* strain noma3 gou, which has been maintained by our research group, was included in the analysis. Monospores of *P. tanegashimensis, P. yezoensis,* and collected samples were cultured in modified SWM-III medium, devoid of the vitamin mixture, Tris buffer, soil extracts, and liver extracts^48^. In this study, oyster shells were artificially seeded to grow conchocelis filaments^49^. The conchocelis were cultivated in an incubator at 20, 25, and 30 °C under illumination (10–20 µmol·m^−2^·s^−1^, 10∶14 h light:dark cycle) with 29–30 psu salinity. The culture medium was replaced weekly and conchosporangia growth was examined under a microscope after 10 days of static culture. Once the conchospores attained an adequate size, the culture was transitioned into an aeration culture, and 10 vinylon filaments were added. The spore release rate was measured by observing the spores attached to filaments under a fluorescence microscope. Finally, leaf area of all samples was measured using Lenaraf 220 b software (Vector Japan Co, Ltd, Japan) (http://www.vector.co.jp/soft/dl/win95/art/se312811.html). Germanium dioxide (5 ppm) was used to eliminate the diatoms in all experiments. All experiment were performed by using three biological replicates.

### Effects of temperature and salinity on the growth of natural spore germlings

All three strains were cultivated at 28 °C under illumination (10–20 µmol·m^−2^ s^−1^, 13∶11 h light:dark cycle). Released spores were cultured at 26, 28, and 30 °C and salinities of 15 and 30 psu in SWM-III medium with Ariake sound supplemented with modified nutrients. The conditions of the released spores were analysed weekly under a microscope according to the above-mentioned procedure. This experiment was performed by referencing a previous study conducted in Myanmar^20^.

### Molecular characterization using *rbcL* sequence

DNA was extracted from the blades of 19 semi dry specimens using the DNAs-ici!-F (RIZO, Japan). To analyse the *rbcL* gene sequences, two primers, Primer_1F (ATGTCTCAATCCGTAGAATCA) and Primer_1R (ATCTTTCCATAAATCTAAAGC), were used for PCR amplification according to the procedure reported in a previous study^50^. The resulting PCR products were purified using ISOSPIN PCR Product (NIPPON GENE, Japan). DNA concentration was measured using the Qubit DNA BR Assay Kit (Thermo Fisher Scientific, CA, USA). To identify the overlapping sequences, four primers, Primer_1F, Primer_1R, Primer_2F (GGAAGATATGTATGARAGAGC), and Primer_2R (GCTCTCYTCATACATATCTTCC), were used to sequence *rbcL* gene. Sequencing was performed at FASMAC (Japan). Closely related species were selected via Basic Local Align Search Tool (BLAST) analysis and the closely related samples used in the analysis are summarised in the Supplementary Table S2. Multiple alignments were performed using the mafft multiple alignment program (version 7.453)^51^. Model test was performed using the MEGA version 11 software^52^. Evolutionary history was inferred using the ML method with a bootstrap value of 500, General Time Reversible model, and Gamma distributed with Invariant Sites (GTR + G + I). All positions with gaps or missing data were eliminated (complete deletion). Analyses were performed using MEGA version 11 software^52^.

### DNA extraction and sequencing using a high-throughput sequencer

DNA was extracted from conchocelis stage of three individuals (*P. vietnamensis* strains MyanmarA, B, and C) using the same extraction method described earlier. Before extraction, DNA was ground to a fine powder using liquid nitrogen. DNA quality was verified via electrophoresis on a 1% agarose gel, and the concentration was measured using the Qubit DNA BR Assay Kit. Sequencing libraries of total DNA were generated by Novogene (Singapore) using the NEBNext Ultra DNA Library Prep Kit for Illumina (NEB, USA). Libraries were sequenced with 150-bp paired-end reads by Novogene using a NovaSeq 6000 instrument (Illumina, USA).

### Molecular characterization using combined dataset of nrSSU and *rbcL* sequences

Using high throughput sequence data, the phylogenetic analysis was performed based on the *rbcL* and nrSSU combined data set, which was provided by Sutherand et al^8^. Adapter sequences and the low-quality bases in the paired-end reads were trimmed using trimmomatic program (v 0.39) with the options (LEADING:20, TRAILING:20, SLIDINGWINDOW:5:20, and MINLEN:50)^53^. The nrSSU rDNA sequences of *Pyropia vietnamensis* strain A and B were obtained using the Getorganelle software^54^ version (v 1.7.3.5) with the following parameters (-R 10 -k 35,85,115 and -F other_pt) and combined with the *rbcL* sequences. The resulting data set was aligned using the mafft multiple alignment program (v7.520)^51^. The appropriate models of sequence evolution were determined using Modeltest-ng (v0.1.7)^55^. Maximum likelihood analysis using the PROTGAMMAILG model was conducted using RAxML (version 8.2.12)^56^ to construct the phylogenetic topology.

### Assembly, gene annotation and molecular analysis of organelle genomes

The cleaned and filtered reads were de novo assembled into organelle genomes using the GetOrganelle toolkit (v 1.7.3.5)^54^ with the following parameters: -R 15, -k 21,45,65,85,105, and-F other_pt. For mitochondrial genome assembly, *Pyropia yezoensis* (DDBJ/EBI/GenBank accession number: JQ736809.1) was used as a seed reference genome with default parameters (-R 30, -k 21,45,65,85,105, -F embplant_mt, and -s seed_sequence). To confirm and modify the assembled results, mapping was performed using Burrows–Wheeler Aligner with default parameters (mem -M -R)^57^ and visualized using the Integrative Genomic Viewer (version 2.11.1)^58^.

Annotations were performed using Geseq (version 2.03)^59^. In this annotation, two sequences (DDBJ/EBI/GenBank accession numbers MK695880 and MK695879) of *P. yezoensis* strain RZ-58^30^ were used as reference genomes for the chloroplast and mitochondrial genomes, respectively. Transfer RNA genes were identified using tRNAscan-SE 1.21^60^. Moreover, ORF was determined using the online tool ORF-Finder program (https://www.ncbi.nlm.nih.gov/orffinder/). Subsequently, the annotation results were corrected manually. Circle genome maps were drawn using the OrganellarGenomeDraw program (version 1.3.1)^61^ with default colouring for gene categories.

To analyse the uncertain regions of mitochondrial genomes using dideoxy sequencing, three pairs of primers, Target_1F (AACGTGGCTACTCGGCTATG) and Target_1R (TCTTTTCCTGGAGCTGCAAT), Target_2F (TTCACAGTATAATTGGGTAATCATTTT) and Target_2R (CAAGTCCGCATGACCCTTAT), and Target_3F (AATTAACCAAACGCCTCACG) and Target_3R (CGCCCTCAACTATGAGTGTTT), were designed for PCR amplification. PCR product purification and quantification were performed as previously described. Sequences of the purified PCR products were determined using a DNA Sequencer (Spectrum Compact CE System (Promega, USA)) with the BigDye Terminator v3.1 kit (Thermo Fisher Scientific, USA).

### Comparative analysis of chloroplast genome sequences

Chloroplast genome sequences of the Myanmar sample, *P. yezoensis* strain RZ-58^30^ (DDBJ/EBI/GenBank accession number: MK695880) and *P. dentata*^33^ (DDBJ/EBI/GenBank accession number: LC521919) were compared using nucleotide BLAST. Nucleotide sequence comparisons of the three genomes were illustrated manually.

### Phylogenetic inference of organelle genomes

Phylogenetic analyses were performed using the assembles organellar genome sequences. The origin of the genome sequences used in the phylogenetic analyses are listed in Supplementary Tables S5 and S6. Multiple alignments with the related sequence datasets were conducted using HomBlocks (version 1.0)^35^ with default parameters. Spurious sequences or poorly aligned regions were removed from multiple sequence alignments using TrimAl (vl.4. rev15)^36^ with the-automated1 setting that was optimized for an ML tree. ModelTest-NG (version 1.7)^55^ was performed to select the best-fit model for evolutionary likelihood. Phylogenetic trees were constructed using Maximum likelihood (ML) and Bayesian inference (BI). For the ML tree, MEGA (version 11)^52^ was used with a general time-reversible model (GTR + I + G4) with a bootstrap value of 500. All positions with gaps or missing data were eliminated (complete deletion). The tree was drawn to scale with branch lengths measured as the number of substitutions per site.

BI phylogenetic tree was constructed using BEAST (v1.10.4)^62^. BEAUti interface was used to generate input files for BEAST, in which the GTR + I + G4 model was applied. A sampling tree was constructed after every 10,000 generations in the Markov Chain Monte Carlo (MCMC) method, which calculates 10,000,000 generations. The first 10% of the generations were eliminated as “burn-in”. The remaining trees were used to construct the consensus tree. To construct phylogenetic tree based on protein sequences, all combination of orthologous proteins were extracted using Orthofinder (v 2.5.4) (-og)^63^. Multiple alignments were performed using MAFFT (v7.520)^51^ and trimmed using trimAL (v1.4.rev15) (automated 1)^36^. ML tree was constructed using IQ-tree (-sp, -bb 1000) (2.2.2.3)^64^. Default parameters were used for all software tools, unless otherwise stated.

### Supplementary Information

Supplementary Information 1.

Supplementary Information 2.

## Data Availability

Chloroplast *rbcL* sequences of the 19 Myanmar seaweeds identified in this study have been deposited in DDBJ/EMBL/GenBank under the accession numbers LC760318 to LC760336. Chloroplast and mitochondrial genome sequences of the collected Myanmar strains A, B, and C have also been deposited in DDBJ/EMBL/GenBank under accession numbers LC761953 to LC761958. The nrSSU sequences were deposited in the DDBJ/EMBL/GenBank under the accession number LC771050 to LC771052. Raw sequencing data for each individual have been deposited in the DDBJ Sequence Read Archive (https://www.ddbj.nig.ac.jp/dra/index-e.html; accession no.DRA015897). The alignments and the tree data used in this study are included as separate files in the Supplementary Dataset.

## References

[1] Yang LE (2020). Redefining *Pyropia* (Bangiales, Rhodophyta): Four new genera, resurrection of *Porphyrella* and description of *Calidia pseudolobata* sp. Nov. from China. J. Phycol..

[2] Mumford Jr, T. F. *Porphyra as Food: Cultivation and Economics in Algae and Human Affairs* (ed. Lembi, C. A. & Waaland, J. R.) 87–117 (Cambridge Univ., 1988).

[3] FAO Fisheries and Aquaculture Information and Statistics Service. *FAO Yearbook: Fishery and Agriculture Statistics 2019* (*United Nations Publs*, 2021).

[4] FAO (Food and Agriculture Organization of the United Nations). The state of world fisheries and aquaculture. http://www.fao.org/3/ca9229en/CA9229EN.pdf, (2020).

[5] Cian RE, Martínez-Augustin O, Drago SR (2012). Bioactive properties of peptides obtained by enzymatic hydrolysis from protein byproducts of *Porphyra columbina*. Food Res. Int..

[6] Cian RE, Garzón AG, Ancona DB, Guerrero LC, Drago SR (2015). Hydrolyzates from P*yropia columbina* seaweed have antiplatelet aggregation, antioxidant and ACE I inhibitory peptides which maintain bioactivity after simulated gastrointestinal digestion. LWT Food Sci. Technol..

[7] Yanagita T, Tsuge K, Koga M, Inoue N, Nagao K (2020). Eicosapentaenoic acid-containing polar lipids from seaweed Susabinori (*Pyropia yezoensis*) alleviate hepatic steatosis in obese db/db mice. Arch. Biochem. Biophys..

[8] Sutherland JE (2011). A new look at an ancient order: Generic revision of the Bangiales (Rhodophyta) 1. J. Phycol..

[9] Guillemin ML (2016). The bladed Bangiales (Rhodophyta) of the South eastern Pacific: Molecular species delimitation reveals extensive diversity. Mol. Phylogenet. Evol..

[10] Kucera H, Saunders GW (2012). A survey of bangiales (rhodophyta) based on multiple molecular markers reveals cryptic diversity 1. J. Phycol..

[11] Sánchez N, Vergés A, Peteiro C, Sutherland JE, Brodie J (2014). Diversity of bladed B angiales (Rhodophyta) in western *M editerranean*: Recognition of the genus *T. hemis* and descriptions of *T. ballesterosii* sp nov, *T. iberica* sp nov, and *Pyropia parva* sp nov. J. Phycol..

[12] Zuccarello GC, Wen X, Kim GH (2022). Splitting blades: Why genera need to be more carefully defined; the case for *Pyropia* (Bangiales, Rhodophyta). Algae.

[13] Santiañez WJE (2020). Proposal of Phycocalidia Santiañez & MJ Wynne nom. Nov. to replace Calidia L. E. Yang & J Brodie nom Illeg. (Bangiales, Rhodophyta). Not Algarum..

[14] Lee JM (2018). Mitochondrial and plastid genomes from coralline red algae provide insights into the incongruent evolutionary histories of organelles. Genome Biol. Evol..

[15] Yang EC (2015). Highly conserved mitochondrial genomes among multicellular red algae of the *Florideophyceae*. Genome Biol. Evol..

[16] Lee J (2016). Parallel evolution of highly conserved plastid genome architecture in red seaweeds and seed plants. BMC Biol..

[17] Muñoz-Gómez SA (2017). The new red algal subphylum Proteorhodophytina comprises the largest and most divergent plastid genomes known. Curr. Biol..

[18] Htun, S. *The Seaweed Resources of Myanmar. Seaweed Resources of the World* 99–105 (*Kanakawa International Fisheries Training Cntr, Japan International Cooperation Agency (JICA)*, 1998).

[19] Thein, M. & Myint, A. *Porphyra crispata* Kjellman (Rhodophyta, Bangiales) from Burma. *In Proceedings of the Burma Research Congress* (1975).

[20] Sein, A. M. Laboratory Culture of the Red Alga, *Porphyra suborbiculata* Kjellman. *Master thesis* in *Mawlamyine University, Myanmar* (1999).

[21] Sein AM (2003). Studies on *Porphyra suborbiculata* Kjellman (Bangiales, Rhodophyta) from Myanmar: I: The morphology and life history in culture. Bull. Mar. Sci. Fish. Kochi Univ..

[22] Tanaka, T. Notes on some marine algae from Viet-Nam-I. *Memoirs of the Faculty of Fisheries, Kagoshima University*, **11,** 21–40 (1962).

[23] Kavale MG, Kazi MA, Sreenadhan N, Singh VV (2015). Morphological, ecological and molecular characterization of *Pyropia* vietnamensis (Bangiales, Rhodophyta) from the Konkan region, India. Phytotaxa.

[24] Tsutsui I (2012). Common underwater plants in coastal areas of Thailand. JIRCAS Int. Agric. Ser..

[25] Milstein D, Medeiros AS, Oliveira EC, Oliveira MC (2015). Native or introduced? A re-evaluation of *Pyropia* species (Bangiales, Rhodophyta) from Brazil based on molecular analyses. Eur. J. Phycol..

[26] Nagano Y (2018). Phylogenetic relationships of Aurantioideae (Rutaceae) based on RAD-Seq. Tree Genet. Genomes.

[27] Yu WB, Huang PH, Li DZ, Wang H (2013). Incongruence between nuclear and chloroplast DNA phylogenies in Pedicularis section Cyathophora (Orobanchaceae). PLoS ONE.

[28] Liang C, Zhang X, Shi L, Hao C, Ye N, Li F (2018). Conserved and novel heat stress-responsive microRNAs identified by deep sequencing in *Pyropia yezoensis*. J. Appl. Phycol..

[29] Xu G, Terada R, Watanabe Y, Nishihara GN (2021). Temperature characteristics on the growth and photosynthesis of a red alga *Phycocalidia tanegashimensis* (= *Pyropia tanegashimensis*, Bangiales) reveal adaptation to subtropical environments due to year-round occurrence of the macroscopic gametophyte. J. Appl. Phycol..

[30] Xu K, Yu X, Tang X, Kong F, Mao Y (2019). Organellar genome variation and genetic diversity of Chinese *Pyropia yezoensis*. Front. Mar. Sci..

[31] Wang L (2013). Complete sequence and analysis of plastid genomes of two economically important red algae: *Pyropia haitanensis* and *Pyropia yezoensis*. PLoS ONE.

[32] Reith M, Munholland J (1995). Complete nucleotide sequence of the*Porphyra purpurea* chloroplast genome. Plant Mol. Biol. Rep..

[33] Choi SJ (2020). Complete chloroplast genome sequences of *Pyropia dentata* (Bangiales, Rhodophyta). Mitochondrial DNA.

[34] Nagano Y, Kimura K, Kobayashi G, Kawamura Y (2021). Genomic diversity of 39 samples of *Pyropia* species grown in Japan. PLoS ONE.

[35] Bi G, Mao Y, Xing Q, Cao M (2018). HomBlocks: A multiple-alignment construction pipeline for organelle phylogenomics based on locally collinear block searching. Genomics.

[36] Capella-Gutiérrez S, Silla-Martínez JM, Gabaldón T (2009). trimAl: A tool for automated alignment trimming in large-scale phylogenetic analyses. Bioinform..

[37] Kim JK (2017). Seaweed aquaculture: Cultivation technologies, challenges and its ecosystem services. Algae.

[38] Yan XH, Lv F, Liu CJ, Zheng YF (2010). Selection and characterization of a high-temperature tolerant strain of *Porphyra haitanensis* Chang et Zheng (Bangiales, Rhodophyta). J. Appl. Phycol..

[39] Lee HJ, Choi J (2019). Enhancing temperature tolerance of *Pyropia tenera* (Bangiales) by inducing mutation. Phycologia.

[40] Li X, Sun X, Gao L, Xu J, Gao G (2021). Effects of periodical dehydration on biomass yield and biochemical composition of the edible red alga *Pyropia yezoensis* grown at different salinities. Algal Res..

[41] Smith DR, Hua J, Lee RW, Keeling PJ (2012). Relative rates of evolution among the three genetic compartments of the red alga *Porphyra* differ from those of green plants and do not correlate with genome architecture. Mol. Phylogenet. Evol..

[42] Kong F, Sun P, Cao M, Wang L, Mao Y (2014). Complete mitochondrial genome of *Pyropia yezoensis*: Reasserting the revision of genus Porphyra. Mitochondrial DNA.

[43] Mao Y, Zhang B, Kong F, Wang L (2012). The complete mitochondrial genome of *Pyropia haitanensis* Chang et Zheng. Mitochondrial DNA.

[44] Foster PG, Hickey DA (1999). Compositional bias may affect both DNA-based and protein-based phylogenetic reconstructions. J. Mol. Evol..

[45] Kainer D, Lanfear R (2015). The effects of partitioning on phylogenetic inference. Mol. Biol. Evol..

[46] Yu G (2022). Data Integration.

[47] Jantzen JR, Whitten WM, Neubig KM, Majure LC, Soltis DE, Soltis PS (2019). Effects of taxon sampling and tree reconstruction methods on phylodiversity metrics. Ecol. Evol..

[48] Ogata E (1970). On a new algal culture medium SWM-III. J. Phycol..

[49] Miura, A. Studies on genetic improvement of cultivated Porphyra (laver). In *Proceedings of the 7th Japan- Soviet Joint Symposium on Aquaculture 161–168* (Tokai Univ, Tokyo, 1979).

[50] Lindstrom SC (2008). Cryptic diversity, biogeography and genetic variation in northeast Pacific species of *Porphyra* sensu lato (Bangiales, Rhodophyta). J Appl. Phycol..

[51] Katoh K, Standley DM (2013). *MAFFT Multiple Sequence Alignment* software version 7: Improvements in Performance and Usability. Mol. Biol. Evol..

[52] Tamura K, Stecher G, Kumar S (2021). MEGA 11: Molecular evolutionary genetics analysis version 11. Mol. Biol. Evol..

[53] Bolger AM, Lohse M, Usadel B (2014). Trimmomatic: A flexible trimmer for Illumina sequence data. Bioinformatics.

[54] Jin JJ (2020). GetOrganelle: A fast and versatile toolkit for accurate de novo assembly of organelle genomes. Genome Biol..

[55] Darriba D (2020). ModelTest-NG: A new and scalable tool for the selection of DNA and protein evolutionary models. Mol. Biol. Evol..

[56] Stamatakis A (2014). RaxML version 8: A tool for phylogenetic analysis and post-analysis of large phylogenies. Bioinformatics.

[57] Li H, Durbin R (2009). Fast and accurate short read alignment with Burrows-Wheeler transform. Bioinformatics.

[58] Robinson JT, Thorvaldsdóttir H, Turner D, Mesirov JP (2023). igv. Js: An embeddable JavaScript implementation of the Integrative Genomics Viewer (IGV). Bioinformatics.

[59] Tillich M (2017). GeSeq: Versatile and accurate annotation of organelle genomes. Nucleic Acids Res..

[60] Schattner P, Brooks AN, Lowe TM (2005). The tRNAscan-SE, snoscan and snoGPS web servers for the detection of tRNAs and snoRNAs. Nucleic Acids Res..

[61] Greiner S, Lehwark P, Bock R (2019). OrganellarGenomeDRAW (OGDRAW) version 1.3.1: Expanded toolkit for the graphical visualization of organellar genomes. Nucleic Acids Res..

[62] Drummond AJ, Suchard MA, Xie D, Rambaut A (2012). Bayesian phylogenetics with BEAUti and the BEAST 1.7. Mol. Biol. Evol..

[63] Emms DM, Kelly S (2019). OrthoFinder: Phylogenetic orthology inference for comparative genomics. Genome Biol..

[64] Nguyen LT, Schmidt HA, Von Haeseler A, Minh BQ (2015). IQ-TREE: A fast and effective stochastic algorithm for estimating maximum-likelihood phylogenies. Mol. Biol. Evol..

