# Supplementary material for: Characterization and organelle genome sequencing of Pyropia species from Myanmar
Source: Sci Rep. 2023 Sep 21;13:15677. doi: 10.1038/s41598-023-42262-3 (PMC10514050; doi:10.1038/s41598-023-42262-3)
Supplement: Supplementary file 1 — Supplementary Information 1. [file 41598_2023_42262_MOESM1_ESM.zip › Supplementary Dataset Legends.docx]

**Supplementary Dataset 1**

Alignment of the RuBisCO LSU (*rbcL*) sequences for inferring Maximum Likelihood tree used Supplementary Fig. S3, in fasta format. (TXT 6kb)

**Supplementary Dataset 2**

Maximum Likelihood tree based on RuBisCO LSU (*rbcL*) sequences (Supplementary Dataset 1), basis for Supplementary Fig. S3, in newick format. (TXT 1kb)

**Supplementary Dataset 3**

Alignment of the combined dataset of concatenated nuclear SSU ribosomal RNA (nrSSU) and RuBisCO LSU (*rbcL*) sequence used for inferring RAxML tree in main text Figure 1, in fasta format. (TXT 43kb)

**Supplementary Dataset 4**

RAxML tree based on combined dataset of concatenated nuclear SSU ribosomal RNA (nrSSU) and RuBisCO LSU (*rbcL*) sequence alignment (Supplemnetary Dataset 3), basic for main text Figure 1, in newick format. (TXT 4kb)

**Supplementary Dataset 5**

Alignment of the combined dataset of concatenated nuclear SSU ribosomal RNA (nrSSU) and RuBisCO LSU (*rbcL*) sequence used for inferring RAxML tree in Supplementary Fig. S4, in fasta format. (TXT 68kb)

**Supplementary Dataset 6**

RAxML tree based on combined dataset of concatenated nuclear SSU ribosomal RNA (nrSSU) and RuBisCO LSU (*rbcL*) sequence alignment (Supplementary Dataset 5), basic for Supplementary Fig. S4, in newick format. (TXT 3kb)

**Supplementary Dataset 7**

Alignment of chloroplast genome sequences used for inferring Maximum Likelihood (ML) tree in main text Figure 7, and Bayesian Inference (BI) in Supplementary Figure 16. (TXT 675kb)

**Supplementary Dataset 8**

Maximum Likelihood (ML) tree based on chloroplast genome sequences alignment (Supplementary Dataset 7), basic for main text Figure 7, in newick format. (TXT 1kb)

**Supplementary Dataset 9**

Bayesian inference (BI) tree based on chloroplast genome sequences alignment (Supplementary Dataset 7), basic for Supplementary Fig. S16, in newick format. (TXT 1kb)

**Supplementary Dataset 10**

Alignment of mitochondria genome sequences used for inferring Maximum Likelihood (ML) tree in main text Figure 8, and Bayesian Inference (BI) in Supplementary Figure 18. (TXT 97kb)

**Supplementary Dataset 11**

Maximum Likelihood (ML)tree based on mitochondria genome sequences alignment (Supplementary Dataset 10), basic for main text Figure 8, in newick format. (TXT 1kb)

**Supplementary Dataset 12**

Bayesian inference (BI) tree based on mitochondria genome sequences alignment (Supplementary Dataset 10), basic for Supplementary Fig. S17, in newick format. (TXT 1kb)

**Supplementary Dataset 13**

Concatenated chloroplast genome protein alignment used for inferring ML tree in Supplementary Fig. S18, in fasta format. (TXT 82kb)

**Supplementary Dataset 14**

Maximum likelihood (ML) tree based on concatenated chloroplast genome protein alignment (Supplementary Dataset 13), basis for Supplementary Fig. S18, in newick format. (TXT 1kb)

**Supplementary Dataset 15**

Concatenated mitochondria genome protein alignment used for inferring ML tree in Supplementary Fig. S18, in fasta format. (TXT 23kb)

**Supplementary Dataset 16**

Maximum likelihood (ML) tree based on concatenated mitochondria genome protein alignment (Supplementary Dataset 15), basis for Supplementary Fig. S18, in newick format. (TXT 6kb)
