# Supplementary material for: Characterization and organelle genome sequencing of Pyropia species from Myanmar
Source: Sci Rep. 2023 Sep 21;13:15677. doi: 10.1038/s41598-023-42262-3 (PMC10514050; doi:10.1038/s41598-023-42262-3)

Characterization and organelle genome sequencing of *Pyropia* species from Myanmar

Myat Htoo San[1,2✉], Yoshio Kawamura[3], Kei Kimura[1,3], Eranga Pawani Witharana[1,4], Takeshi Shimogiri[1,5], San San Aye[6], Thu Thu Min[7], Cherry Aung[8], Moe Moe Khaing[9], Yukio Nagano[1,2,4✉]

[1] *The United Graduate School of Agricultural Sciences, Kagoshima University, Kagoshima, Japan*

[2] *Analytical Research Center for Experimental Sciences, Saga University, Saga, Japan*

[3] *Faculty of Agriculture, Saga University, Saga, Japan*

[4] *Graduate School of Advanced Health Science, Saga University, Saga, Japan*

[5] *Faculty of Agriculture, Kagoshima University, Kagoshima, Japan*

[6] *Mawlamyine University, Mawlamyine, Myanmar*

[7] *Marine Science Department, Pathein University, Pathein, Myanmar*

[8] *Marine Science Department, Myeik University, Myeik, Myanmar*

[9] *Botany Department, Dawei University, Dawei, Myanmar*

***Corresponding author:**

✉E-mail: k1747459@kadai.jp

✉E-mail: nagano@cc.saga-u.ac.jp

Supplementary Table S1: Basionym, homotypic synonyms and current classification of the species involved in this study.

Basionym	Homotypic Synonyms	Current Classification
Monostroma endiviifolium, Gepp, A. & Gepp, E.S. Antarctic algae. *Journal of Botany, British and Foreign* **43**: 105-109, pl. 470, (1905).		*Pyropia endiviifolia* (A.Gepp & E.Gepp) H.G.Choi & M.S.Hwang, in Sutherland, J.E., Lindstrom, S.C., Nelson, W.A., Brodie, J., Lynch, M.D., Hwang, M.S. et al. A new look at an ancient order: generic revision of the Bangiales (Rhodophyta). *Journal of Phycology* 47(5): 1131-1151 (2011)
Porphra kanakaensis, Mumford, T.F., Jr, A new species of *Porphyra* from the west coast of North America. *Syesis 6*: 239-242, 8 figs (1973).		*Pyropia kanakaensis* (Mumford) S.C.Lindstrom in Sutherland, J.E., Lindstrom, S.C., Nelson, W.A., Brodie, J., Lynch, M.D., Hwang, M.S. *et al*. A new look at an ancient order: generic revision of the Bangiales (Rhodophyta). *Journal of Phycology* **47(5):** 1131-1151 (2011)
Porphyra pulchra, Smith, G. M., & Hollenberg, G. J. On some Rhodophyceae from the Monterey Peninsula, California. *American journal of botany*, 211-222 (1943).	*Porphyra pulchra* (Hollenberg) T.J.Farr & J.E.Sutherland, 2011 Sutherland, J.E., Lindstrom, S.C., Nelson, W.A., Brodie, J., Lynch, M.D., Hwang, M.S. *et al.* A new look at an ancient order: generic revision of the Bangiales (Rhodophyta). *Journal of Phycology* **47(5):** 1131-1151 (2011)	*Pyropia pulchra* (Hollenberg) S.C.Lindstrom & Hughey, in Lindstrom, S.C. & Hughey, J.R. *Pyropia smithii* is *Pyropia pulchra* comb. nov. *Madroño* **63(3)**: 281-282, 1 fig (2016).
Porphyra perforata, Agardh, J. G. Till algernes systematik. Nya bidrag (Tredje afdelningen). *Lunds Univ. ArsskrÅrsskr., Afd. Math. och Naturvet*, ***19*****(2)** (1883).	*Pyropia perforata* (J.Agardh) S.C.Lindstrom, 2011 Sutherland, J.E., Lindstrom, S.C., Nelson, W.A., Brodie, J., Lynch, M.D., Hwang, M.S. *et al.* A new look at an ancient order: generic revision of the Bangiales (Rhodophyta). *Journal of Phycology* **47(5):** 1131-1151 (2011)	*Neoporphyra perforata* (J.Agardh) L.-E.Yang & J.Brodie comb.nov in Yang, L.-E., Deng, Y.-Y., Xu, G.-P., Russel, S., Lu, Q.-Q. & Brodie, J. Redefining *Pyropia* (Bangiales, Rhodophyta): four new genera, resurrection of *Porphyrella* and description of *Calidia pseudolobata* sp. nov. from China. *Journal of Phycology* **56(4):** [1-18] 862-879, 4 figs (2020).
Pyropia dentata Kjellman, F. R. Japanska arter af slagtet Porphyra. *Bihang K. Sv. Vet.-Akad. Handl. Afd. 3*, *23*, 1-34 (1897).	*Pyropia dentata* (Kjellman) N.Kikuchi & M.Miyata, 2011 Sutherland, J.E., Lindstrom, S.C., Nelson, W.A., Brodie, J., Lynch, M.D., Hwang, M.S. *et al.* A new look at an ancient order: generic revision of the Bangiales (Rhodophyta). *Journal of Phycology* **47(5):** 1131-1151 (2011)	*Neoporphyra dentata* (Kjellman) L.-E.Yang & J.Brodie, 2020 in Yang, L.-E., Deng, Y.-Y., Xu, G.-P., Russel, S., Lu, Q.-Q. & Brodie, J. Redefining *Pyropia* (Bangiales, Rhodophyta): four new genera, resurrection of *Porphyrella* and description of *Calidia pseudolobata* sp. nov. from China. *Journal of*

		Phycology **56(4):** [1-18] 862-879, 4 figs (2020).
Porphyra haitanensis, Chang, T.J. & Zheng, B.F. *Porphyra haitenensis*, a new species of *Porphyra* from Fukien. *Acta Botanica Sinica* **9(1):** 32-36, 5 plates (1960).	*Pyropia haitanensis* (T.J.Chang & B.F.Zheng) N.Kikuchi & M.Miyata, 2011 Sutherland, J.E., Lindstrom, S.C., Nelson, W.A., Brodie, J., Lynch, M.D., Hwang, M.S. *et al.* A new look at an ancient order: generic revision of the Bangiales (Rhodophyta). *Journal of Phycology* **47(5):** 1131-1151 (2011)	*Neoporphyra haitanensis* (T.J.Chang & B.F.Zheng) J.Brodie & L.-E.Yang, 2020 in Yang, L.-E., Deng, Y.-Y., Xu, G.-P., Russel, S., Lu, Q.-Q. & Brodie, J. Redefining *Pyropia* (Bangiales, Rhodophyta): four new genera, resurrection of *Porphyrella* and description of *Calidia pseudolobata* sp. nov. from China. *Journal of Phycology* **56(4):** [1-18] 862-879, 4 figs (2020).
Porphyra fucicola, Krishnamurthy, V. A revision of the species of the algal genus *Porphyra* occurring on the Pacific coast of North America. *Pacific Science* **26:** 24-49 (1972).	*Pyropia fucicola* (V.Krishnamurthy) S.C.Lindstrom, 2011 Sutherland, J.E., Lindstrom, S.C., Nelson, W.A., Brodie, J., Lynch, M.D., Hwang, M.S. *et al.* A new look at an ancient order: generic revision of the Bangiales (Rhodophyta). *Journal of Phycology* **47(5):** 1131-1151 (2011)	*Neopyropia fucicola* (V.Krishnamurthy) L.-E.Yang & J.Brodie, 2020 in Yang, L.-E., Deng, Y.-Y., Xu, G.-P., Russel, S., Lu, Q.-Q. & Brodie, J. Redefining *Pyropia* (Bangiales, Rhodophyta): four new genera, resurrection of *Porphyrella* and description of *Calidia pseudolobata* sp. nov. from China. *Journal of Phycology* **56(4):** [1-18] 862-879, 4 figs (2020).
Porphyra yezoensis, Ueda, S. Systematic study of the genus *Porphyra* in Japan. *Suiko-Kenkyu-Kokoku* **28:** 1-45, 24 tables (1932).	*Pyropia yezoensis* (Ueda) M.S.Hwang & H.G.Choi, 2011 Sutherland, J.E., Lindstrom, S.C., Nelson, W.A., Brodie, J., Lynch, M.D., Hwang, M.S. *et al.* A new look at an ancient order: generic revision of the Bangiales (Rhodophyta). *Journal of Phycology* **47(5):** 1131-1151 (2011)	*Neopyropia yezoensis* (Ueda) L.-E.Yang & J.Brodie, 2020 in Yang, L.-E., Deng, Y.-Y., Xu, G.-P., Russel, S., Lu, Q.-Q. & Brodie, J. Redefining *Pyropia* (Bangiales, Rhodophyta): four new genera, resurrection of *Porphyrella* and description of *Calidia pseudolobata* sp. nov. from China. *Journal of Phycology* **56(4):** [1-18] 862-879, 4 figs (2020).
Porphyra umbilicalis Kützing, F. T. *Phycologia generalis oder Anatomie, Physiologie und Systemkunde der Tange: Textbd* (Vol. 1). Brockhaus (1843).		*Porphyra umbilicalis* **Kützing(1843)** in Kützing, F. T. *Phycologia generalis oder Anatomie, Physiologie und Systemkunde der Tange: Textbd* (Vol. 1). Brockhaus (1843).
Porphyra schizophylla Smith, G. M., & Hollenberg, G. J. On some Rhodophyceae from the Monterey Peninsula, California. *American journal of botany,* 211-222 (1943).		*Wildemania schizophylla* (Hollenberg) S.C.Lindstrom, 2011 Sutherland, J.E., Lindstrom, S.C., Nelson, W.A., Brodie, J., Lynch, M.D., Hwang, M.S. *et al.* A new look at an ancient order: generic revision of the Bangiales (Rhodophyta). *Journal of Phycology* **47(5):** 1131-1151 (2011)
Conferva fuscopurpurea Dillwyn, L. W. *British Confervæ: or, colored figures and descriptions of the British plants referred by*		*Bangia fuscopurpurea* (Dillwyn) Lyngbye, 1819 in Lyngbye, H. C. *Tentamen hydrophytologiae danicae, continens omnia hydrophyta cryptogama Daniae,*

botanists to the genus Conferva. W. phillips (1809).		*Holsatiae, Faeroae, Islandiae, Groenlandiae hucusque cognita, systematice disposita, descripta et iconibus illustrata, adjectis simul speciebus norvegicis: opus praemio in universitate regia Havniensi ornamentum, sumtu regio editum* (Vol. 1). Gyldendal (1819).
Pyropia nitida Harden, L.K., Morales, K.M. & Hughey, J.R. Identification of a new marine algal species Pyropia nitida sp. nov (Bangiales: Rhodophyta) from Monterey, *California. Mitochondrial DNA* 27: 3058-3062 (2015).		*Pyropia nitida* Harden, L.K., Morales, K.M. & Hughey, J.R (2015) in Harden, L.K., Morales, K.M. & Hughey, J.R. Identification of a new marine algal species *Pyropia nitida* sp. nov (Bangiales: Rhodophyta) from Monterey, *California. Mitochondrial DNA* 27: 3058-3062 (2015).
Porphyra crispata Kjellman, F. R. Japanska arter af slagtet Porphyra. *Bihang K. Sv. Vet.-Akad. Handl. Afd.* ***3*****,** *23*, 1-34 (1897).		*Porphyra crispata* Kjellman, 1897 in Kjellman, F. R. Japanska arter af slagtet Porphyra. *Bihang K. Sv. Vet.-Akad. Handl. Afd.* ***3*****,** *23*, 1-34 (1897).
Porphyra tenera Kjellman, F. R. Japanska arter af slagtet Porphyra. *Bihang K. Sv. Vet.-Akad. Handl. Afd.* ***3*****,** *23*, 1-34 (1897).	*Pyropia tenera* (Kjellm.) N. Kikuchi, M. Miyata, M. S. Hwang et H. G. Choi in Sutherland, J.E., Lindstrom, S.C., Nelson, W.A., Brodie, J., Lynch, M.D., Hwang, M.S. *et al.* A new look at an ancient order: generic revision of the Bangiales (Rhodophyta). *Journal of Phycology* **47(5):** 1131-1151 (2011)	*Neopyropia tenera (Kjellm.)* L.-E.Yang & J.Brodie, 2020 in Yang, L.-E., Deng, Y.-Y., Xu, G.-P., Russel, S., Lu, Q.-Q. & Brodie, J. Redefining *Pyropia* (Bangiales, Rhodophyta): four new genera, resurrection of *Porphyrella* and description of *Calidia pseudolobata* sp. nov. from China. *Journal of Phycology* **56(4):** [1-18] 862-879, 4 figs (2020).
Porphyra tanegashimensis Shinmura, I. *Porphyra tanegashimensis,* a new species of Rodophyceae from Tanegashima Island in southern Japan. *Bulletin..* (1974).	*Calidia tanegashimensis* (Shinmura) L.-E.Yang & J.Brodie, 2020 in Yang, L.-E., Deng, Y.-Y., Xu, G.-P., Russel, S., Lu, Q.-Q. & Brodie, J. Redefining *Pyropia* (Bangiales, Rhodophyta): four new genera, resurrection of *Porphyrella* and description of *Calidia pseudolobata* sp. nov. from China. *Journal of Phycology* **56(4):** [1-18] 862-879, 4 figs (2020).	*Phycocalidia tanegashimensis* (I.Shinmura) Santiañez, 2020 in Santiañez, W.J.E. Proposal of *Phycocalidia* Santiañez & MJ Wynne nom. nov. to replace *Calidia* L. E. Yang & J. Brodie nom. illeg.(Bangiales, Rhodophyta). *Not Algarum,* 140, pp.1-3 (2020)
Porphyra vietnamensis Tanaka, T. & Pham-Hoang, H. Notes of some marine algae from Viet-Nam - I. *Memoirs of the Faculty of Fisheries, Kagoshima University* 11: 24-40 (1962).	*Calidia vietnamensis* (Tak.Tanaka & P.H.Hô) L.-E.Yang & J.Brodie**,** 2020 in Yang, L.-E., Deng, Y.-Y., Xu, G.-P., Russel, S., Lu, Q.-Q. & Brodie, J. Redefining *Pyropia* (Bangiales, Rhodophyta): four new genera, resurrection of *Porphyrella* and description of *Calidia pseudolobata* sp. nov. from China. *Journal of Phycology* **56(4):** [1-18] 862-879, 4 figs (2020).	*Phycocalidia vietnamensis* (I.Shinmura) Santiañez, 2020 in Santiañez, W.J.E. Proposal of *Phycocalidia* Santiañez & MJ Wynne nom. nov. to replace *Calidia* L. E. Yang & J. Brodie nom. illeg.(Bangiales, Rhodophyta). *Not Algarum,* 140, pp.1-3 (2020)

	Pyropia vietnamensis (Tak. Tanaka & P.H.Ho) J.E.Sutherland & Monotilla, 2011<br>Sutherland, J.E., Lindstrom, S.C., Nelson, W.A., Brodie, J., Lynch, M.D., Hwang, M.S. *et al.* A new look at an ancient order: generic revision of the Bangiales (Rhodophyta). *Journal of Phycology* **47(5):** 1131-1151 (2011)	
Porphyra suborbiculata Kjellman, *Bihang til Kongliga Svenska Vetenskaps-Akademiens Handlingar,* Afd. III 23(4): 10, pl. 1: figs 1-3; pl. 2: figs 5-9; pl. 5: figs 4-7 (1897)	*Phyllona suborbiculata* (Kjellman) Kuntze, *Revisio generum plantarum. Pars III (3):* 420 (1898)<br><br>*Pyropia suborbiculata* (Kjellman) Sutherland, J.E., Lindstrom, S.C., Nelson, W.A., Brodie, J., Lynch, M.D., Hwang, M.S. *et al.* A new look at an ancient order: generic revision of the Bangiales (Rhodophyta). *Journal of Phycology* **47(5):** 1131-1151 (2011)<br><br>*Calidia suborbiculata* (Kjellman) L.-E.Yang & J.Brodie in Yang et al., *Journal of Phycology*<br>56(3): [5], 2020, nom. illeg.	*Phycocalidia suborbiculata* (Kjellman) Santiañez & M.J.Wynne, *comb. Nov*<br>Santiañez, W.J.E. Proposal of *Phycocalidia* Santiañez & MJ Wynne nom. nov. to replace *Calidia* L. E. Yang & J. Brodie nom. illeg.(Bangiales, Rhodophyta). *Not Algarum,* 140, pp.1-3 (2020)

Supplementary Table S2. List of *rbcL* sequences used in the phylogenetic analysis.

Species Name	Accession Number
Pyropia vietnamensis	HQ687544.1
Pyropia sp. "Piaui"	HQ605697.1
Pyropua denticulata	HQ687521.1
Porphra tanegashimensis	LC434505.1
Pyropia acanthophora	KY272468.1
Pyropia yamadae	LC328315.1
Pyropia suborbiculata	LC434500.1
Pyropia gardneri	JN028971.1
Pyropia elongata	FJ817088.1
Pyropia koreana	KJ561211.1
Pyropia tenera	AB366148.1
Pyropia yezoensis	MK695880.1
Pyropia dentata	LC521919.1
Pyropia spiralis	HQ605696.1
Pyropia seriata	LC505533.1
Pyropia pseudolinearis	AB287929.1
Pyropia pulchra	NC029861.1
Pyropia protolanceolata	KP904006.1
Pyropia lanceolata	KP904039.1
Pyropia pseudolanceolata	KP904058.1
Uedaea onoi	MG926664.1
Porphyra purpurea	NC000925.1

Supplementary Table S3. List of raw data generated from high-throughput sequencing

Strain name	Raw data amount (Gb)	Reads
P. vietnamensis strain MyanmarA	10.4	69,507,106
P. vietnamensis strain MyanmarB	15.2	101,123,102
P. vietnamensis strain MyanmarC	11.9	79,121,346

Supplementary Table S4. Information on rRNA clusters in the chloroplast genome used in phylogenetic analysis. "_" represents the absence of genes, "✓" represents the presence of genes, and "O" represents the partial presence of genes. The direction of all rRNA clusters is the same.

Species names	rrsA	rrlA	rrfA	rrsB	rrlB	rrfB
Pyropia endiviifolia	✓	✓	✓	✓	✓	✓
Pyropua kanakaensis	✓	✓	✓	✓	✓	✓
Porphra pulchra	✓	✓	✓	✓	✓	✓
Pyropia perforate	✓	✓	✓	✓	✓	✓
Pyropia denata	✓	✓	✓	**O**	**O**	✓
Pyropia haitanensis	✓	✓	✓	✓	✓	✓
Pyropia fucoila	✓	✓	✓	✓	✓	✓
Pyropia yezoensis	✓	✓	✓	✓	✓	✓
Wildermanian schizophyla	✓	✓	✓	✓	✓	✓
Bangia fuscopurpurea	✓	✓	✓	✓	✓	✓
Porphyra umbilicalis	✓	✓	✓	✓	✓	✓
Pyropia vietnamensis strain MyanmarA	✓	✓	✓	–	–	–
Pyropia vietnamensis strain MyanmarB	✓	✓	✓	–	–	–

Supplementary Table S5. List of chloroplast genome sequences used in the phylogenetic analysis.

Species Name	Accession Number
Pyropia endiviifolia	KT716756.1
Pyropua kanakaensis	KJ776836.1
Porphra pulchra	KT266789.1
Pyropia perforate	KJ776833.1
Pyropia denata	LLC521919.1
Pyropia haitanensis	KC464603.1
Pyropia fucicola	KJ776837.1
Pyropia yezoensis	MK695889.1
Wildermanian schizophyla	KR020505.1
Bangia fuscopurpurea	KP714733.1
Porphyra umbilicalis	MF385003.1

Supplementary Table S6. List of mitochondrial genome sequences used in the phylogenetic analysis.

Species Name	Accession Number
Pyropia kanakaensis	KJ708765.1
Pyropia nitida	KP890080.1
Pyropia endiviifolia	KU356193.1
Pyropia pulchra	MT588076.1
Pyropia perforata	KJ708768.1
Pyropia crispate	MW822759.1
Pyropia haitanensis	JQ736808.1
Pyropia fucicola	KJ708762.1
Pyropua tenera	KC750917.1
Pyropia yezoensis	MK695879.1
Bangia fuscopurpura	KP710691.1
Wildemania schizophylla	KJ689442.1
Porphyra umbilicalis	NC018544.1

Supplementary Fig. S1. Location of the collection site in Myanmar. The collection area is indicated by green squares, while marine areas are depicted in blue. The black circle indicates precise location of the collection site of *Pyropia* samples. The figure was generated using SimpleMappr (https://www.simplemappr.net).

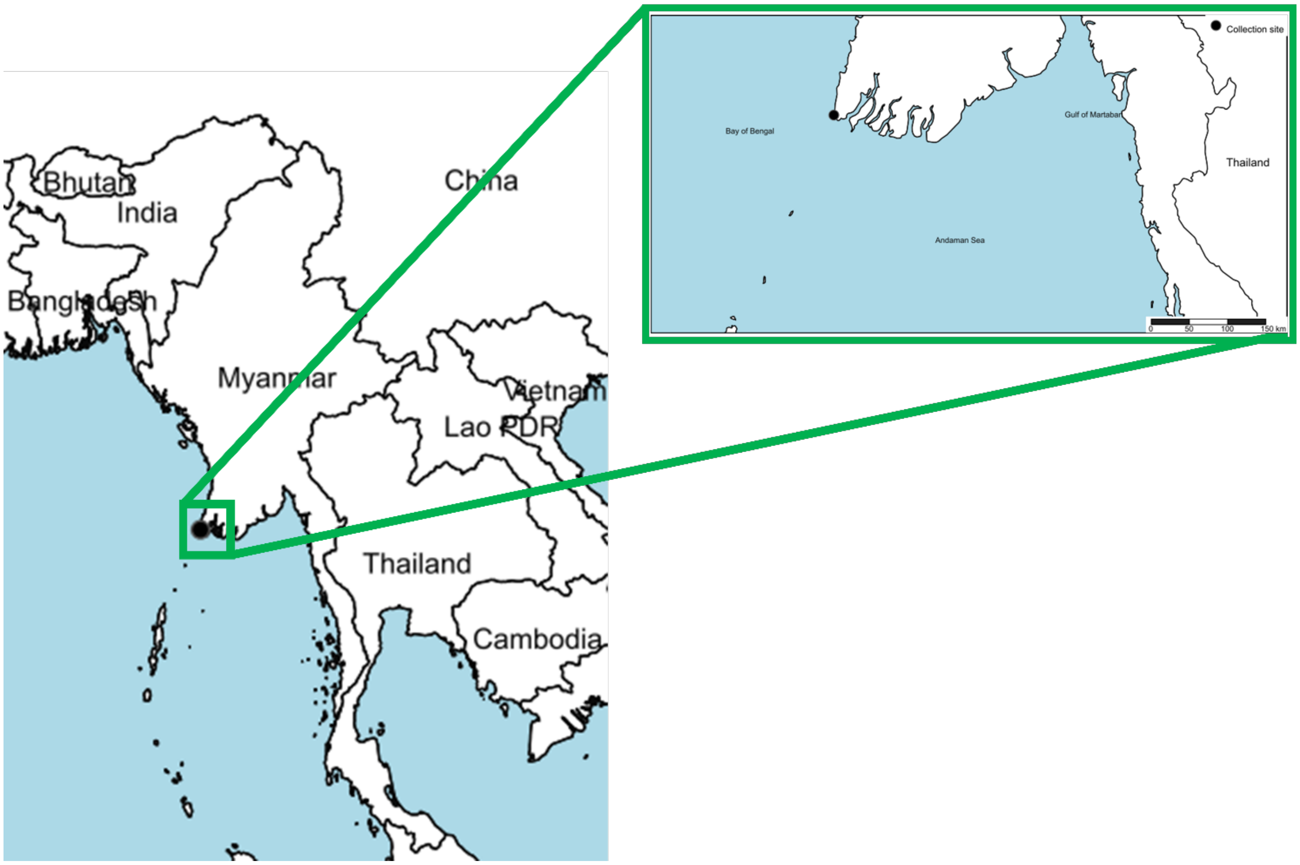

Supplementary Fig. S2. Morphologies of three strains: *Pyropia vietnamensis* strain Myanmar A (on the left), *Pyropia vietnamensis* strain Myanmar B (in the middle), and *Pyropia vietnamensis* strain Myanmar C (on the right). The top photographs depict the three strains after sample collection, while the bottom photographs show herbarium specimens after laboratory culture.

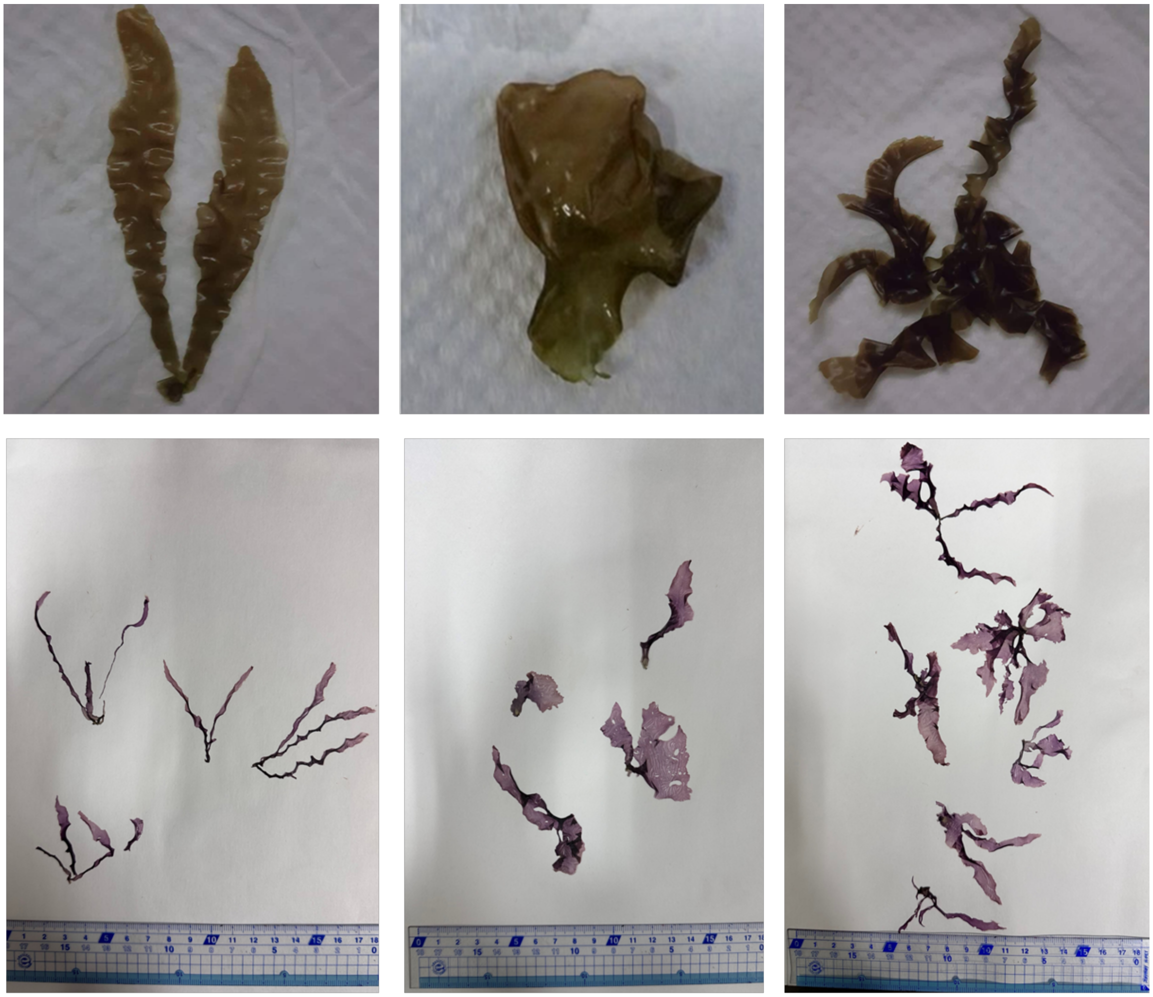

Supplementary Fig. S3. Molecular characterization based on *rbcL* sequences**.** The origin of the samples used in this analysis are described in Supplementary Table 2. Totally 1,961 nucleotide positions (300 parsimony informative sites) were used in this analysis. The alignment data is available in fasta format as Supplementary Dataset 1, and ML tree is available in newick format as Supplementary Dataset 2. Clade names used in the figure are those described by Yang et al.[1].

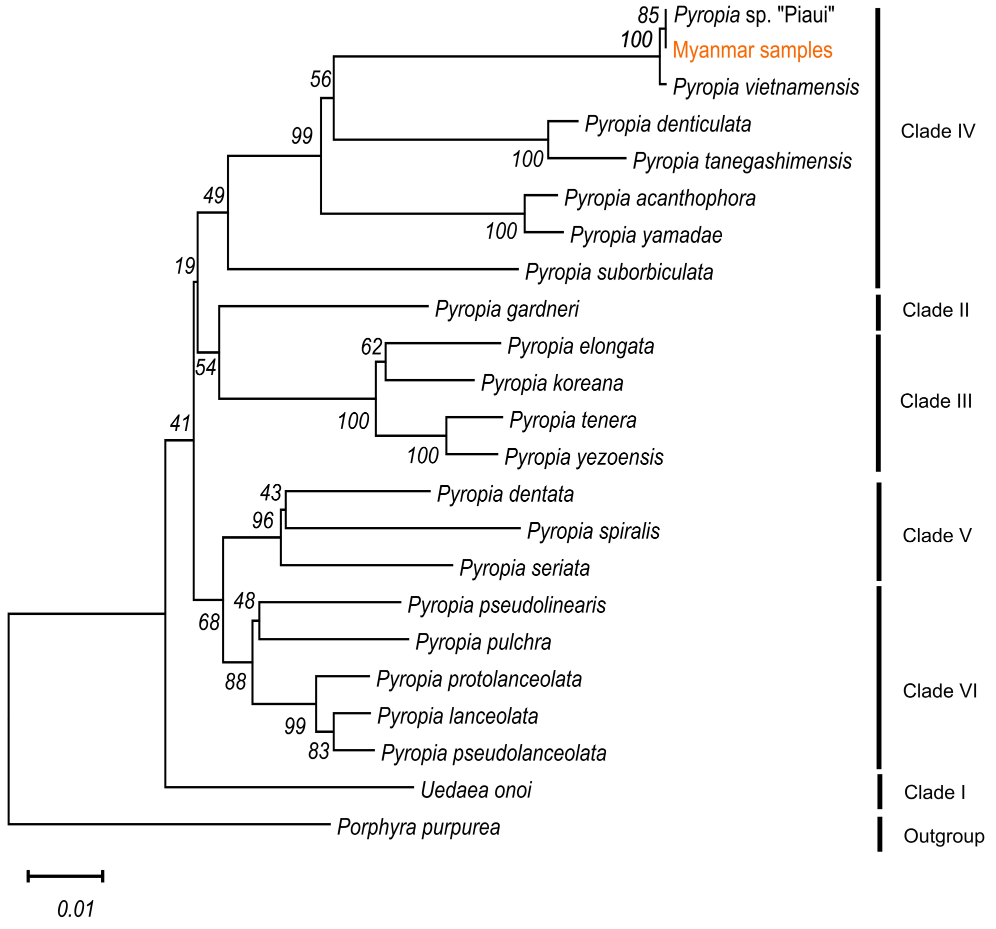

Supplementary Fig. S4. Maximum-likeihood phylogram of Bangiales taxa calculated from the concantenated nucear SSU ribosomal RNA (nrSSU) and RuBisCO LSU (*rbcL*) data from Myanmar specimens and the dataset described by Sutherland at al.,[13] set (TreeBASE ID:S11223) under RAxML. The numbers at the nodes indicate bootstrap values (% over 500 replicates). Scale bar indicates number of substitutions per site. Samples from Myanmar was shown by orange color. The clade names used in the figure are those described by Yang et al.[1]. Total 3,430 nucleotide positions (1,148 parsimony informative sites) were used in this analysis. The alignment data is available in fasta format as Supplementary Dataset 5, and ML tree is available in newick format as Supplementary Dataset 6.

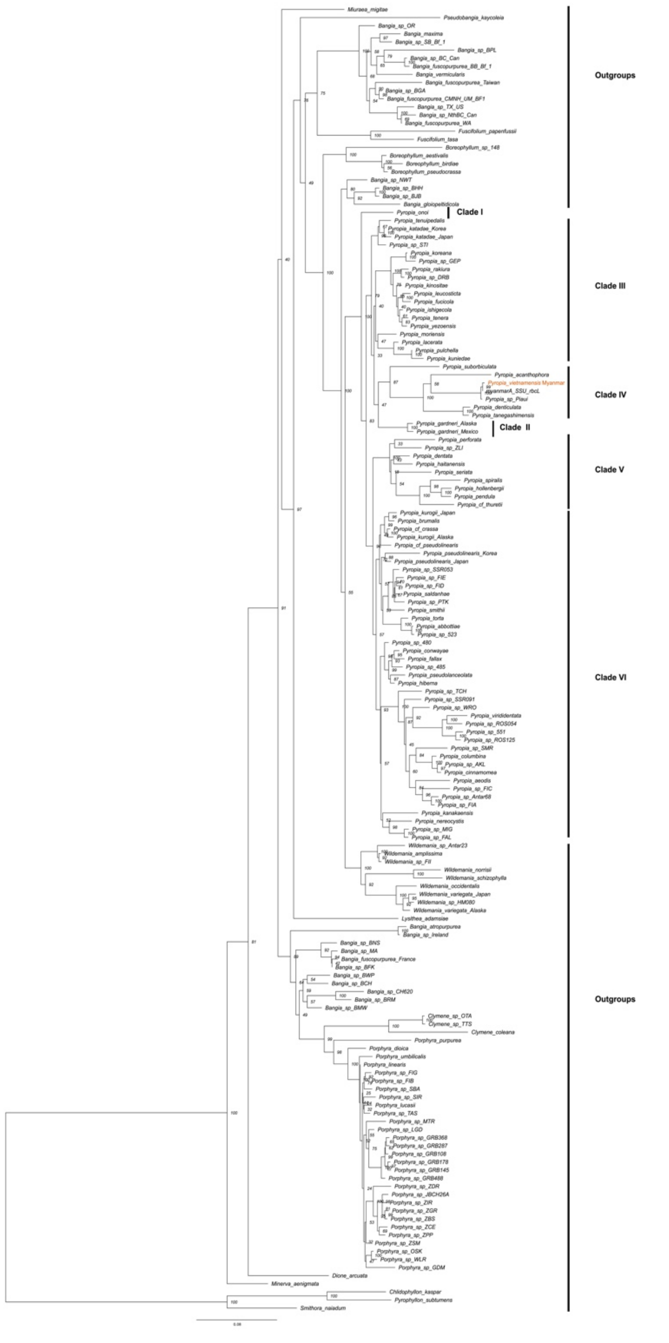

Supplementary Fig. S5. Conchocelis growth of 1) *Pyropia vietnamensis* strain MyanmarA, 2) *Pyropia vietnamensis* strain MyanmarB, 3) *Pyropia vietnamensis* strain MyanmarC, 4) *Pyropia yezoensis* and 5) *Pyropia tanegashimensis* at 20°C, 25°C and 30°C.

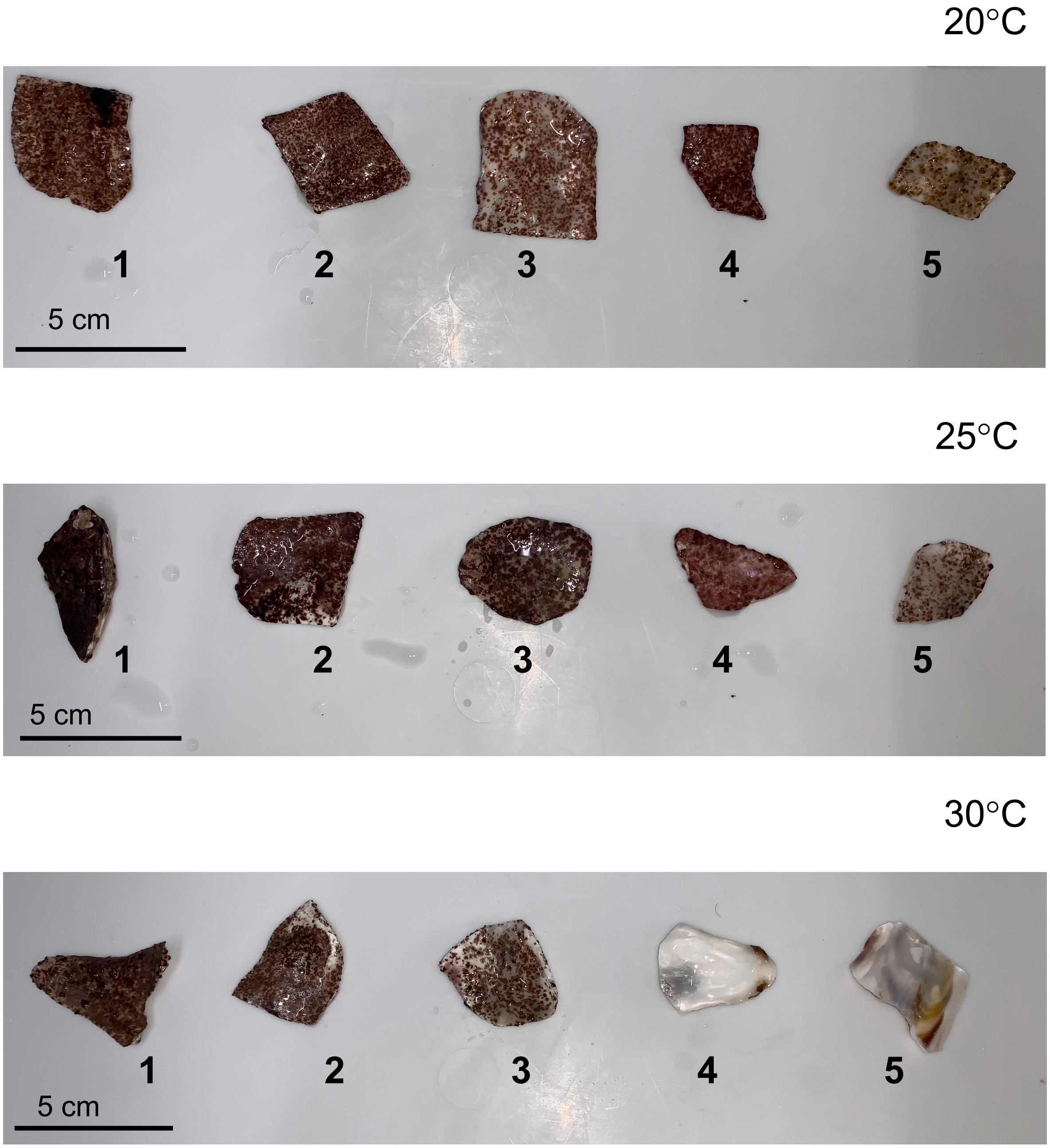

Supplementary Fig. S6. Microscopic observation of conchocelis growth at the temperature of 20°C, 25°C, and 30°C.There were no differences at this stage.

Pyropia vietnamensis strain MyanmarA

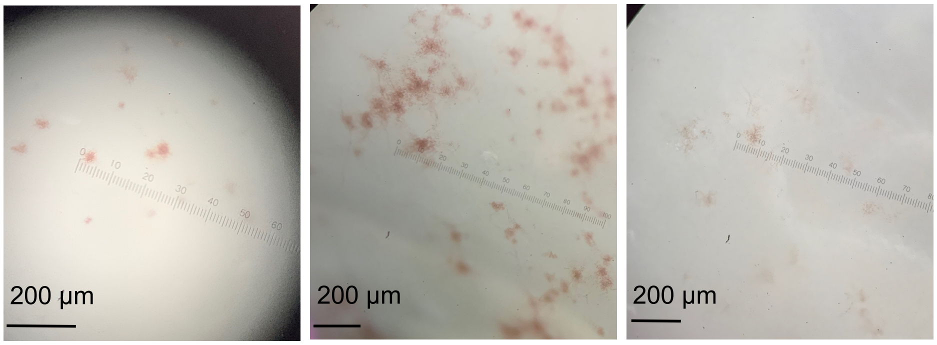

20°C 25°C 30°C

Pyropia vietnamensis strain MyanmarB

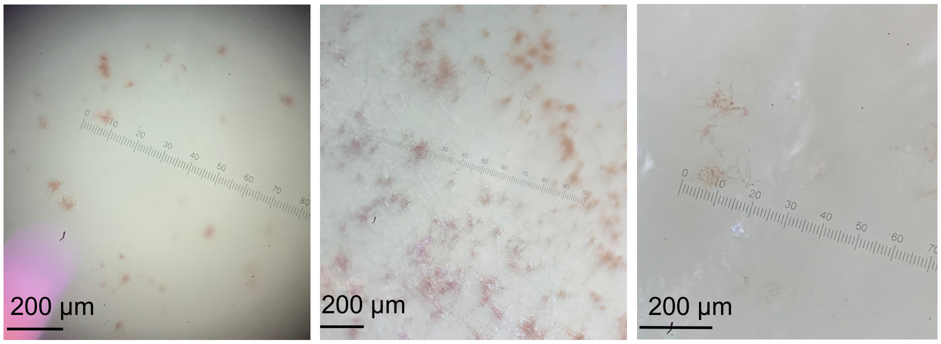

20°C 25°C 30°C

Pyropia vietnamensis strain MyanmarC

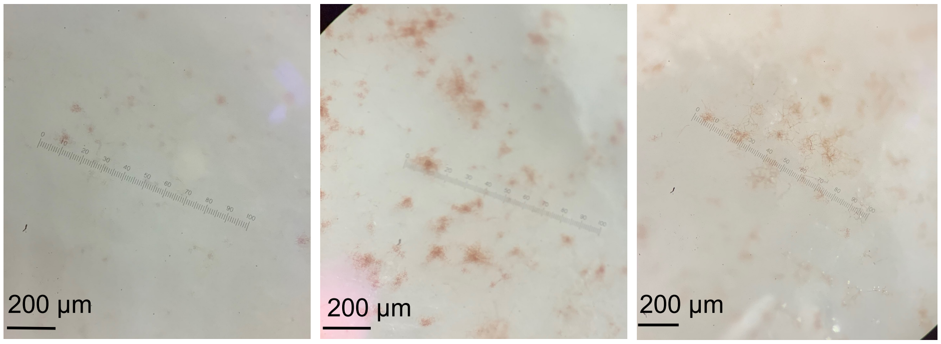

20°C 25°C 30°C

Pyropia yezoensis strain noma3

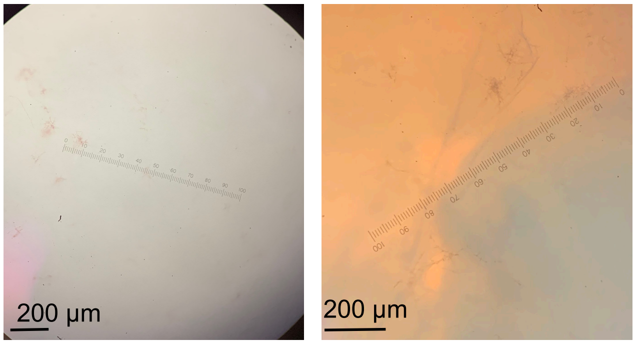

20°C 25°C

Pyropia tanegashimensis

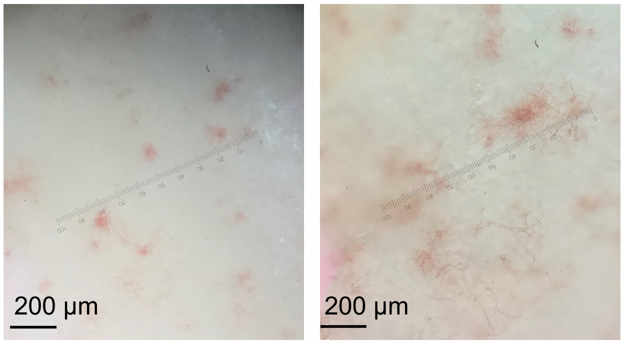

20°C 25°C

Supplementary Fig. S7. Average number of conchosporangia at 20°C (**A**), 25°C (**B**), and 30°C (**C**). Error bars represent standard deviation of triplicate cultures.

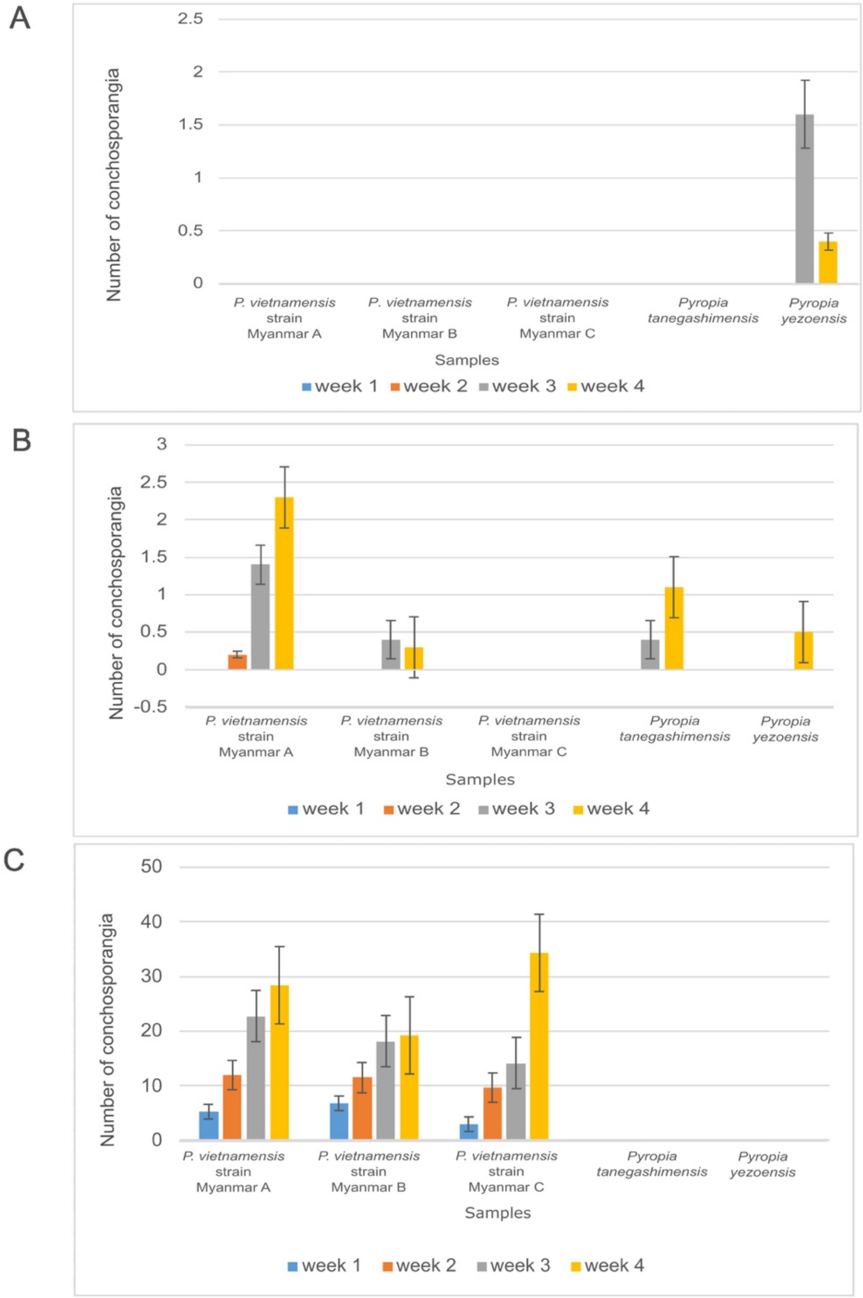

Supplementary Fig. S8. Average number of released natural spore germlings at the temperature of 28°C (**A**) and 25°C (**B**). No release was observed at the temperature of 20°C. Error bars represent standard deviation of triplicate cultures.

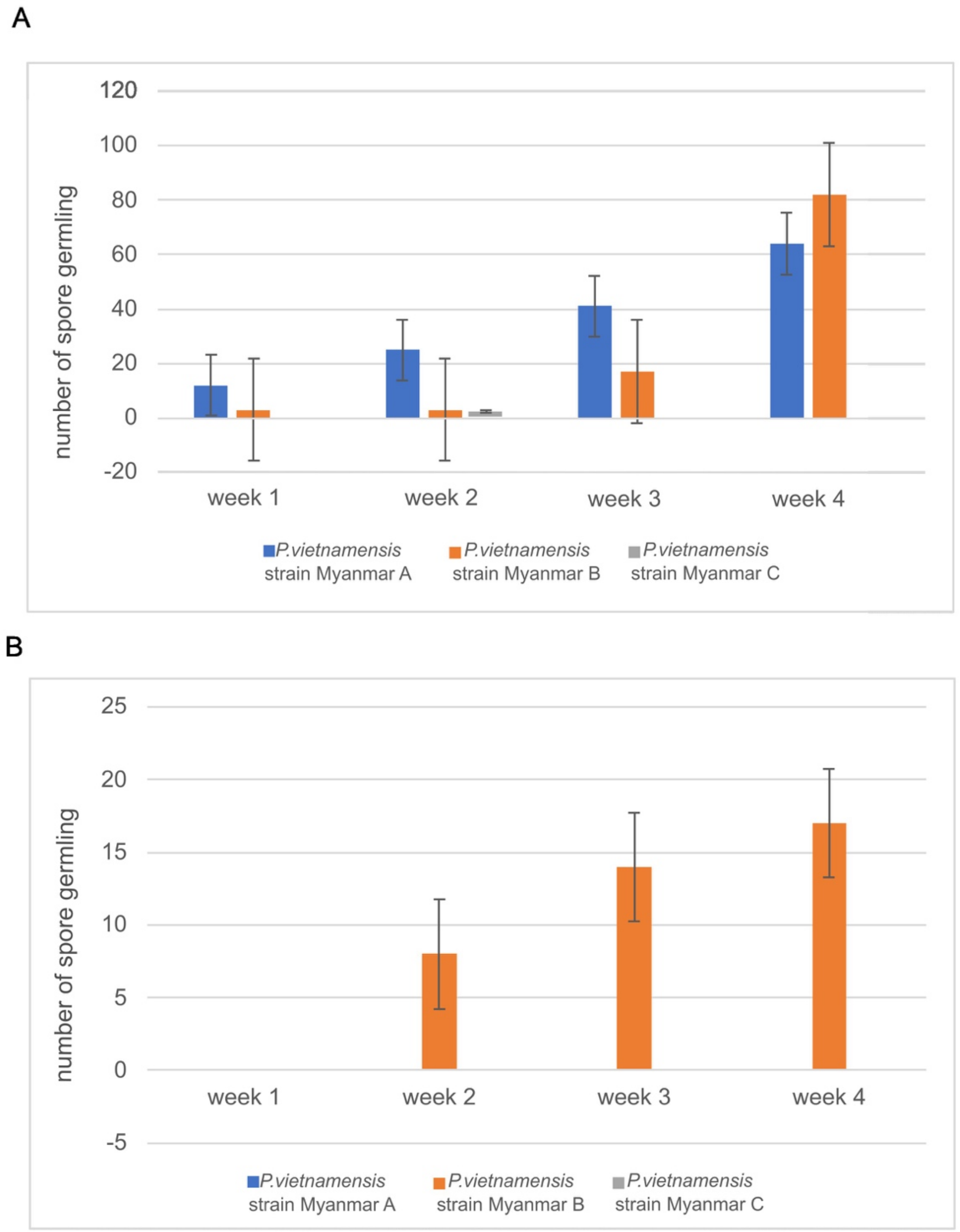

Supplementary Fig. S9. The weekly growth rate of *Pyropia vietnamensis* strain Myanmar A at the temperature of 20°C, 25°C, and 28°C. Error bars represent standard deviation of triplicate cultures.

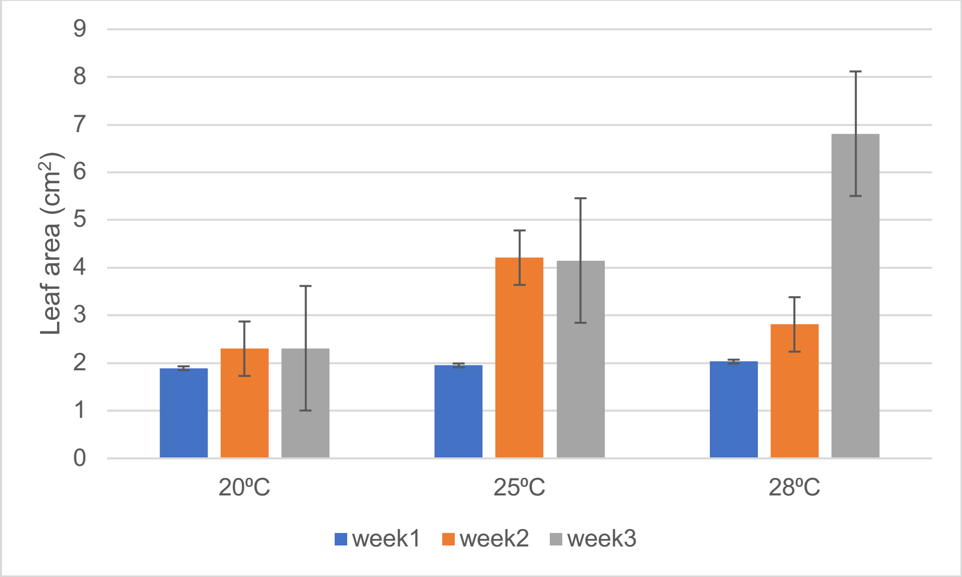

Supplementary Fig. S10. Monecious characteristics of cultured *Pyropia vietnamensis* strain

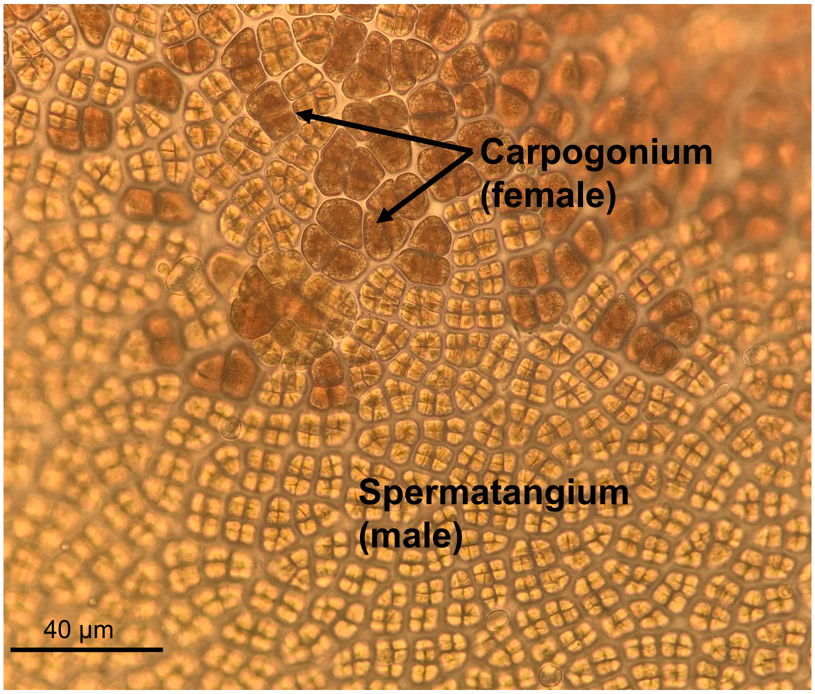

Supplementary Fig. S11. IGV view of the mapping result of the chloroplast genome of three Myanmar species: **A**) *Pyropia vietnamensis* strain Myanmar A, **B**) *Pyropia vietnamensis* strain Myanmar B, and **C**) *Pyropia vietnamensis* strain Myanmar C.

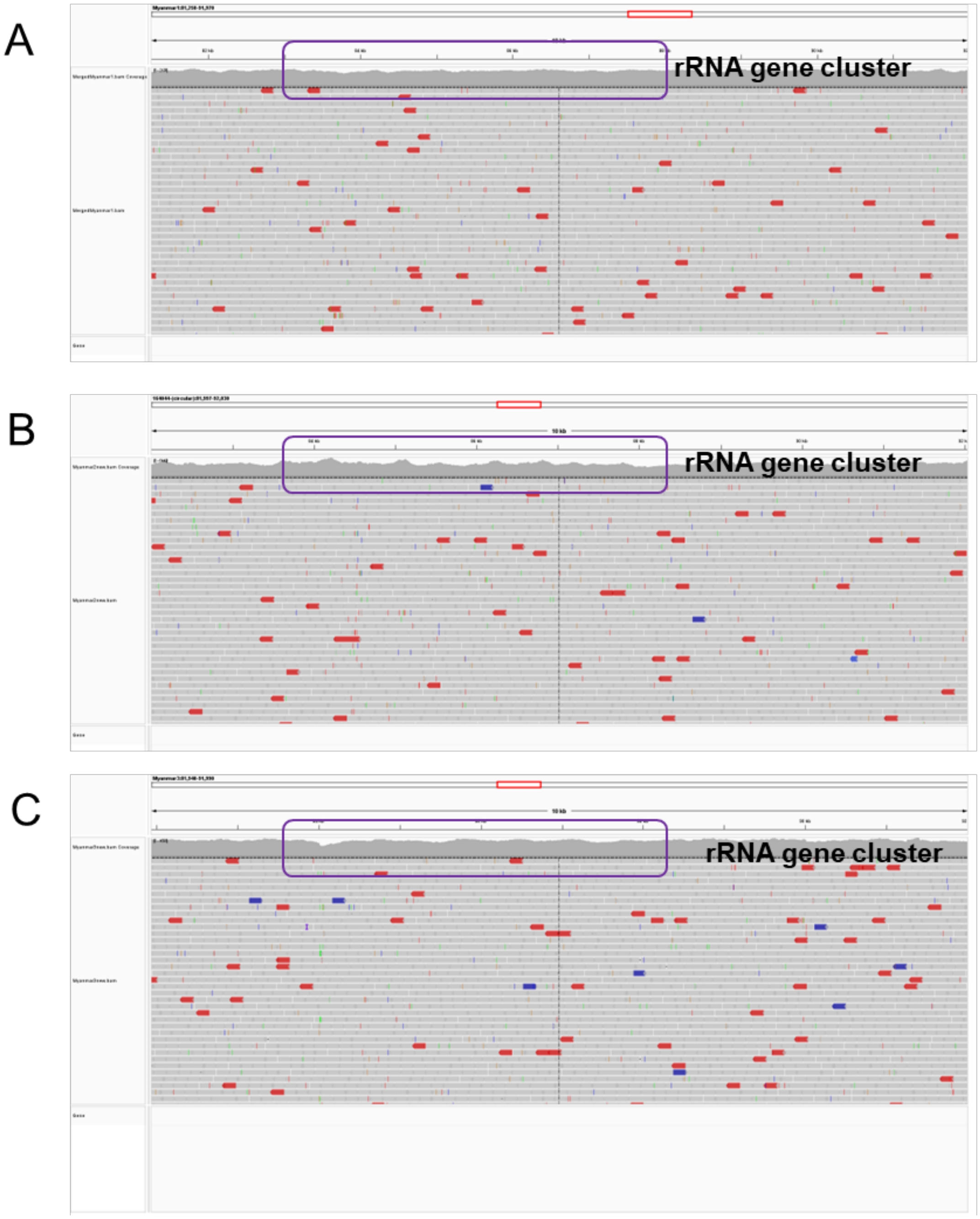

Supplementary Fig. S12. Physical map of the chloroplast genome of *Pyropia vietnamensis* strain Myanmar B created using OrganellerGenomeDraw (OGDRAW; ogdraw.mpimp-golm.mpg.de). Genes are colored according to their function. Anticlockwise transcribed genes are on the outer side and clockwise transcribed genes are on the innnerside of the circle. Dashed area in the inner circle indicates the guanine-cytosine (GC) content of the organelle genomes.

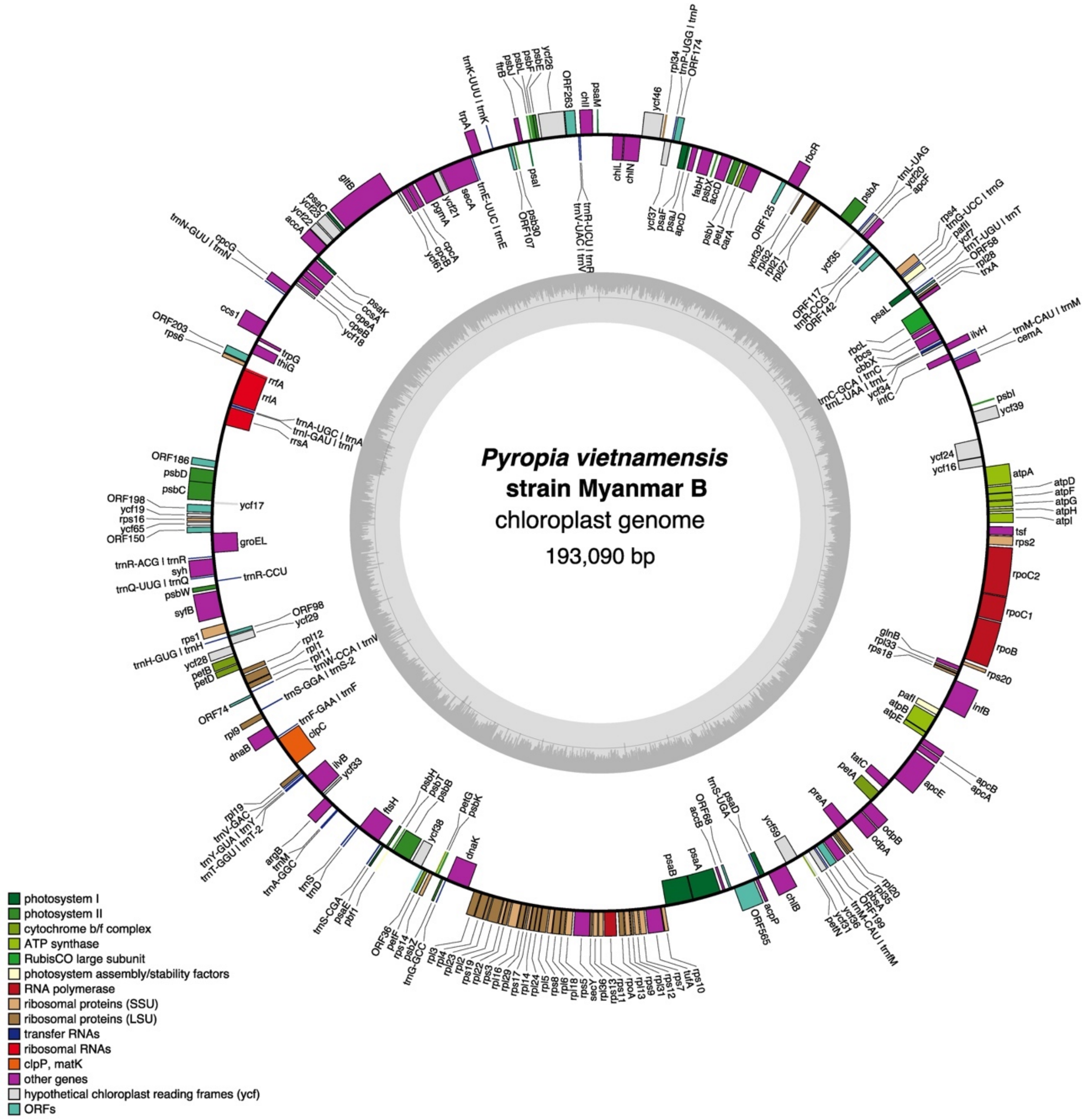

Supplementary Fig. S13. Dot plot result of the comparison between two identical sequences. The figure displays the results of the chloroplast genome sequence comparison of (**A**) the Japanese species *Pyropia yezoensis*, (**B**) the Korean species *Pyropia denata*, and (**C**) the Myanmar species, *Pyropia vietnamensis*. The red circle indicates the presence of the rRNA repeat region. The figure was generated using the dot plot function of BLAST.

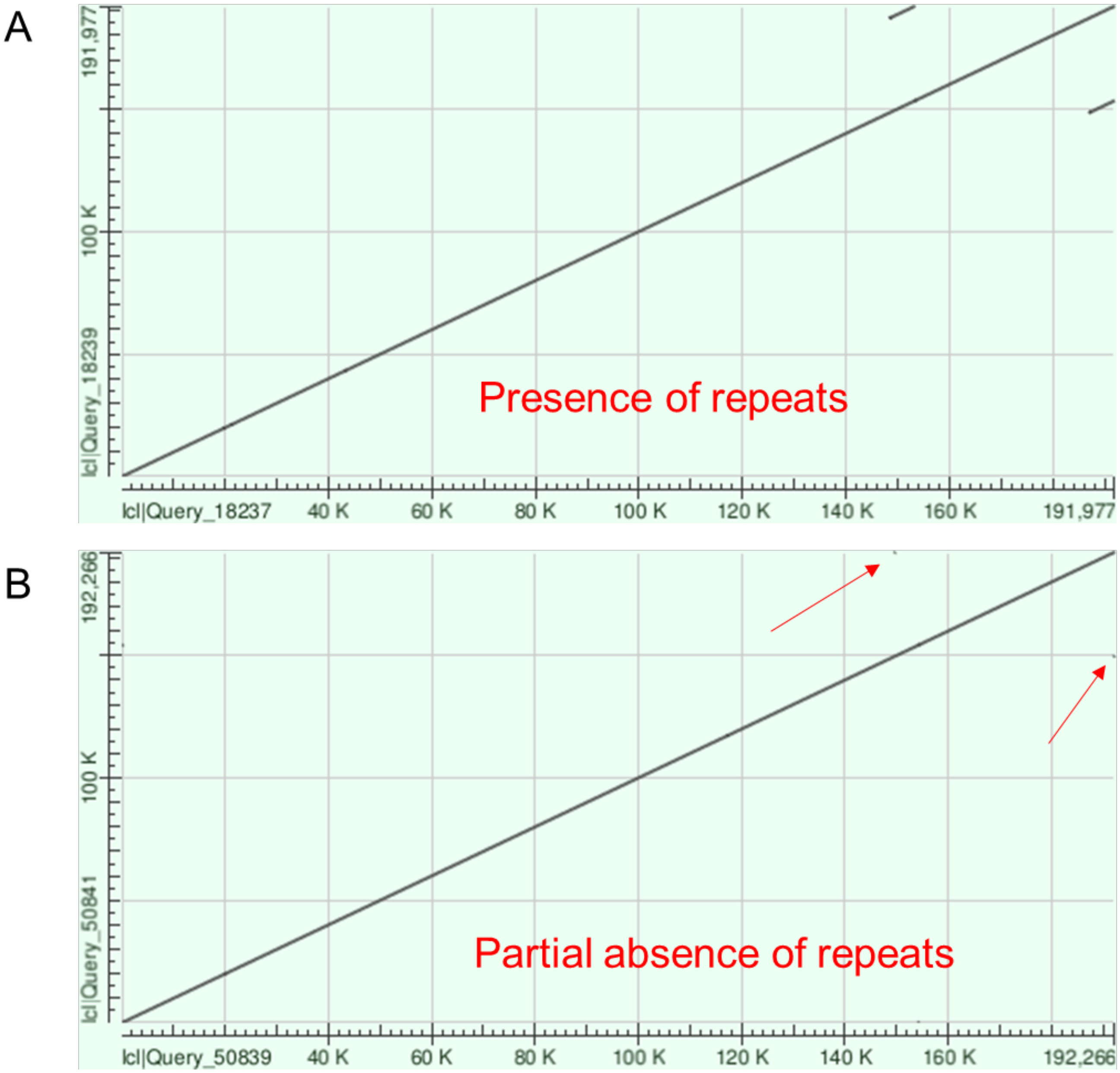

C

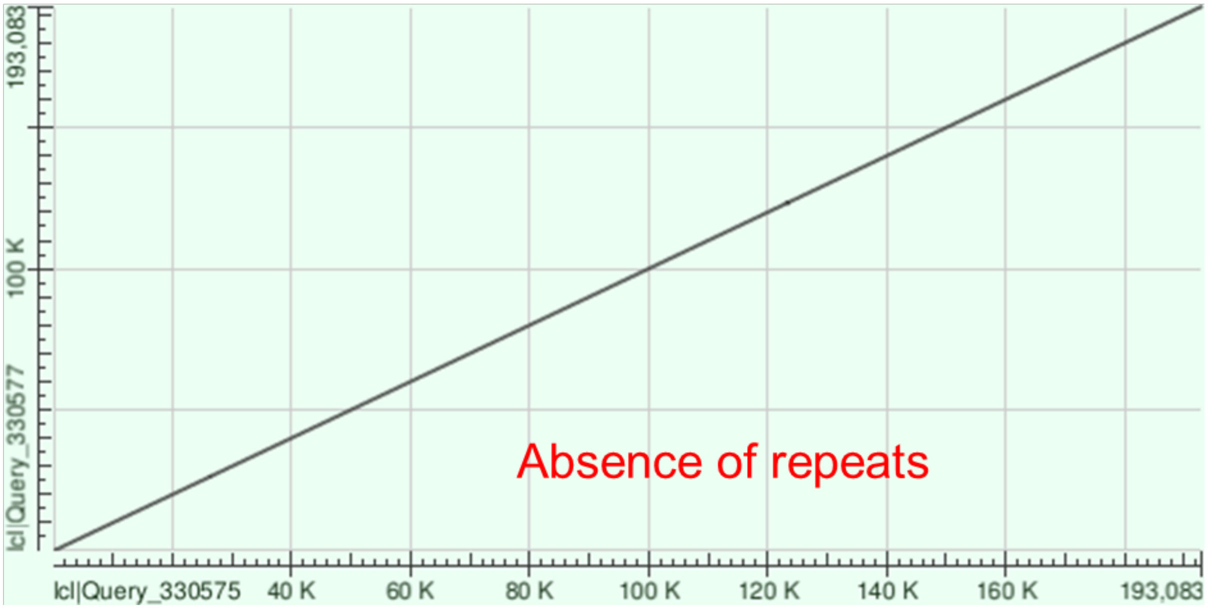
193,083
100 K
lcl|Query_330577
lcl|Query_330575
40 K
60 K
80 K
100 K
120 K
140 K
160 K
193,083
Absence of repeats

Supplementary Fig. S14. IGV investigation of the mapping results among the three forms of Myanmar species, *Pyropia vietnamensis*. Figure (**A**) shows the mapping results of form A, Figure (**B**) shows the mapping results of form B, and Figure (**C**) shows the mapping results of form C.

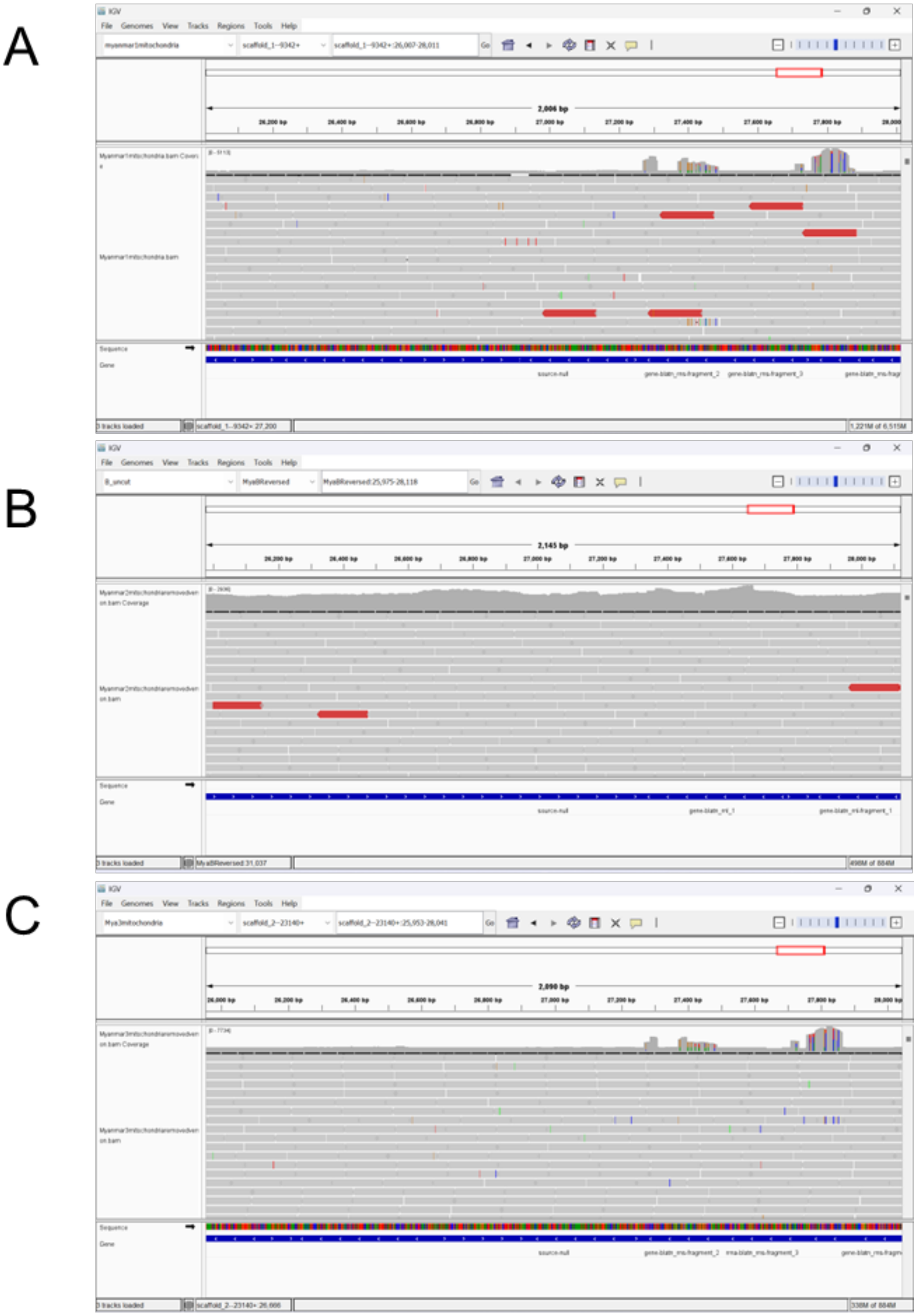

Supplementary Fig. S15. Physical map of the mitohcondrial genome of Myanmar seaweed, *Pyropia vietnamensis* strain Myanmar B, created using OrganellerGenomeDraw (OGDRAW; ogdraw.mpimp-golm.mpg.de). Genes are colored according to their function. Anticlockwise transcribed genes are on the outer side and clockwise transcribed genes are on the inner side of the circle. Dashed area in the inner circle indicates the guanine-cytosine (GC) content of the organelle genomes.

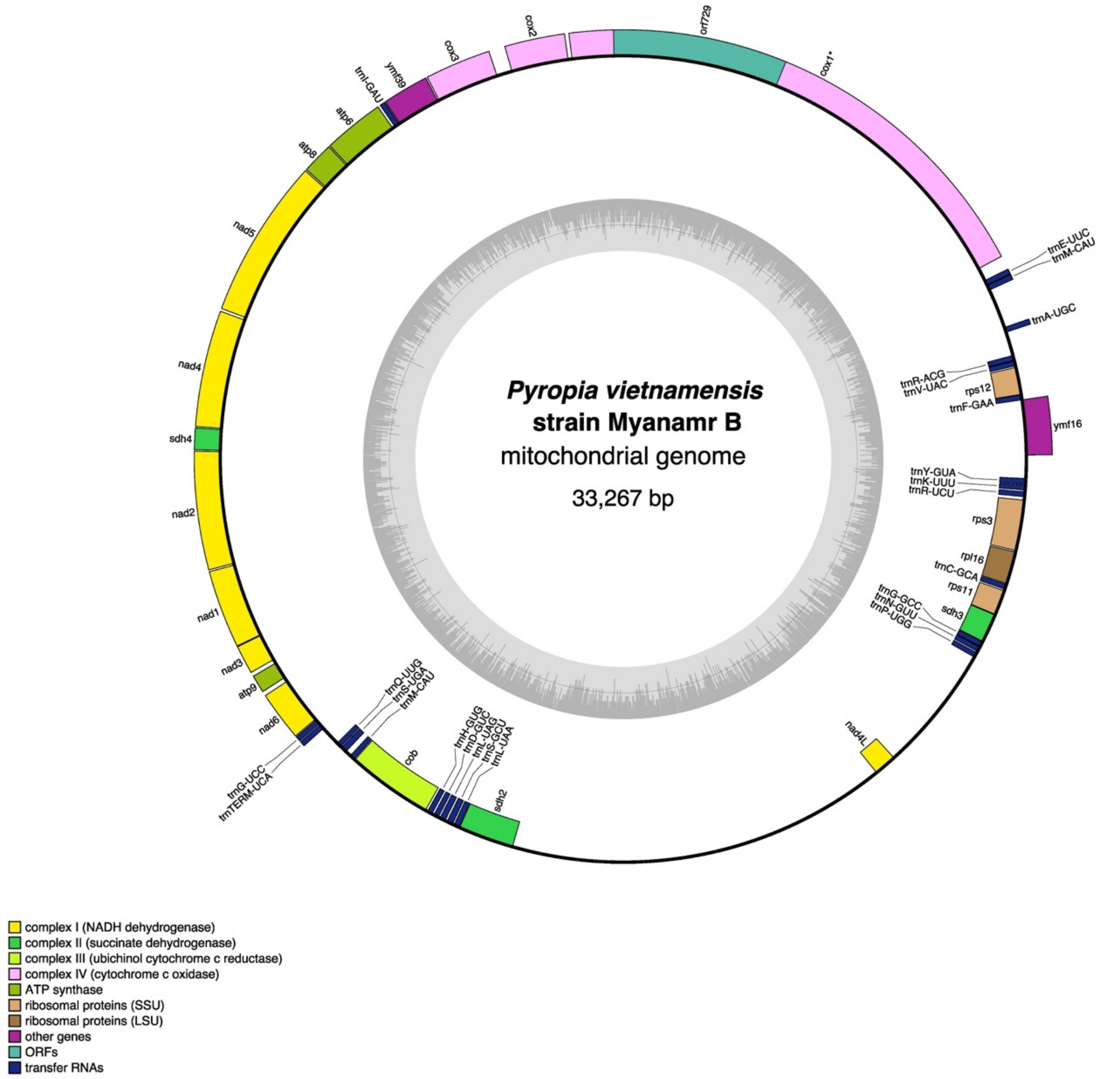

Supplementary Fig. S16. Bayesian inference (BI) tree of chloroplast genomes of *Pyropia* species. BI tree was constructed using the chloroplast genome dataset and Myanmar seaweed and available sequence data on the database. BI support values are presented at each node. Totally 166,616 nucleotide positions (44,834 parsimony informative sites) were used in this analysis. The origin of the samples used in this analysis are described in Supplementary Table 5. The alignment data is available in fasta format as Supplementary Dataset 7, and BI tree is available in newick format as Supplementary Dataset 9.

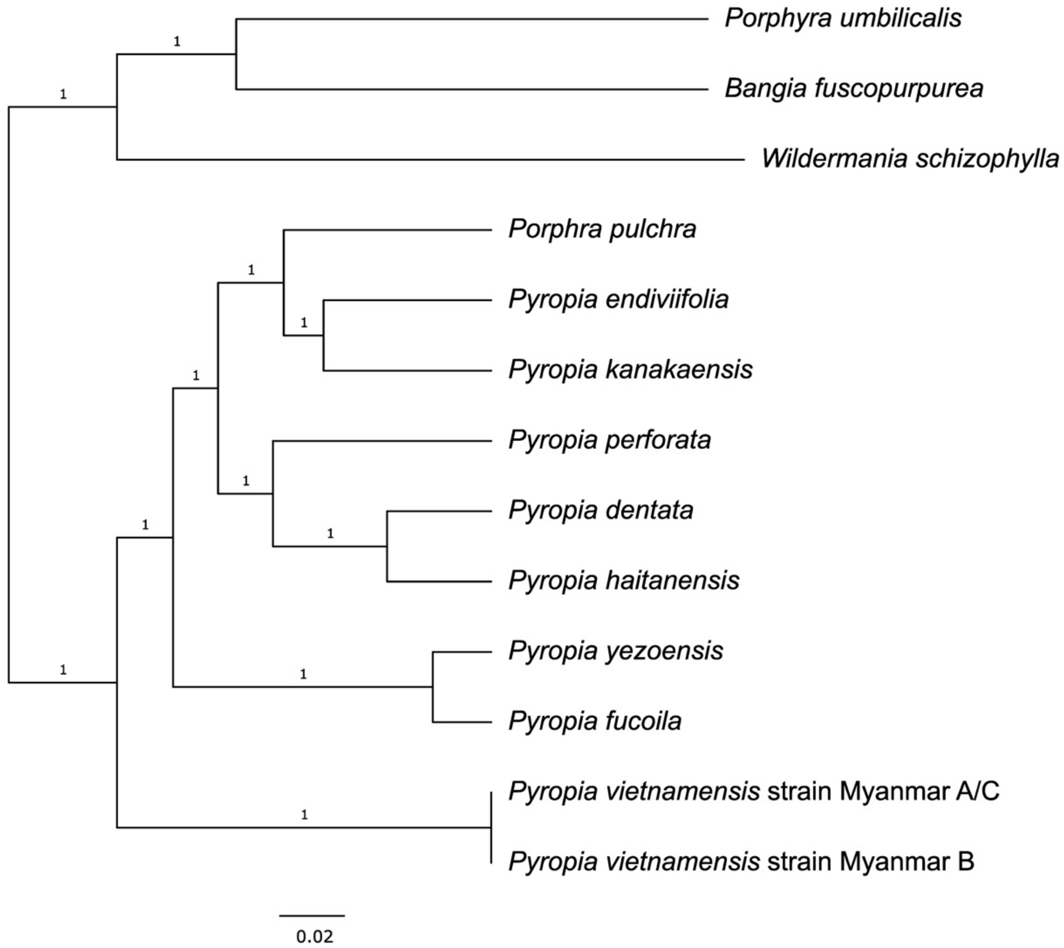

Supplementary Fig. S17. Bayesian inference (BI) tree of mitochondria genomes of *Pyropia* species. BI tree was constructed using the mitochondria genome dataset and Myanmar seaweed and available sequence data on the database. BI support values are presented at each node. Totally 23,066 nucleotide positions (7,007 parsimony informative sites) were used in this analysis. The origin of the samples used in this analysis are described in Supplementary Table 6. The alignment data is available in fasta format as Supplementary Dataset 10, and BI tree is available in newick format as Supplementary Dataset 12.

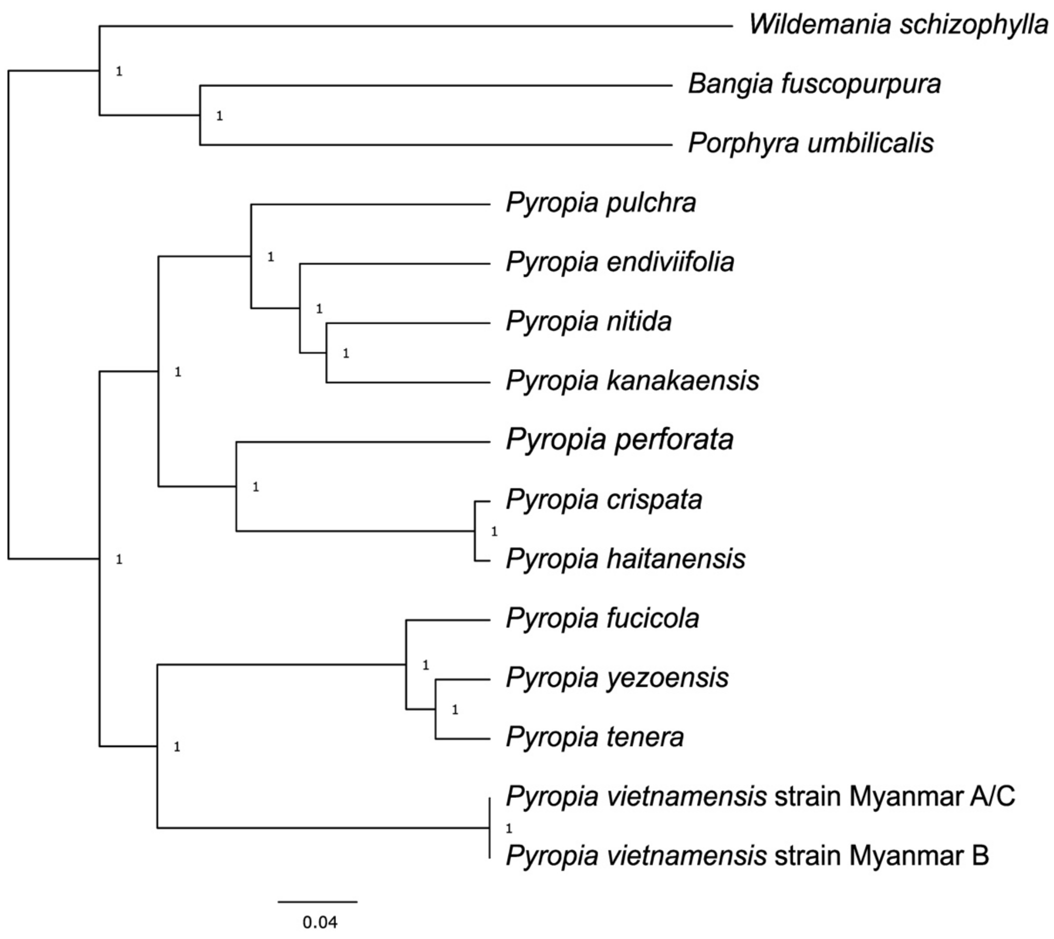

Supplementary Fig. S18. Phylogenetic tree of the chloroplast genome of *Pyropia* species. A maximum likelihood (ML) tree was constructed using the chloroplast genome dataset of Myanmar seaweed and available sequence data on the database. ML bootstrap support values are presented at each node. Orange colour indicates the loss of the repeat region in the sample. The clade names used in the figure are those described by Yang et al.[1]. A total of 166,616 nucleotide positions (44,834 parsimony informative sites) were used in this analysis. The origin of the samples used in this analysis is described in Supplementary Table 5. The alignment data is available in fasta format as Supplementary Dataset 7, and ML tree is available in newick format as Supplementary Dataset 8.

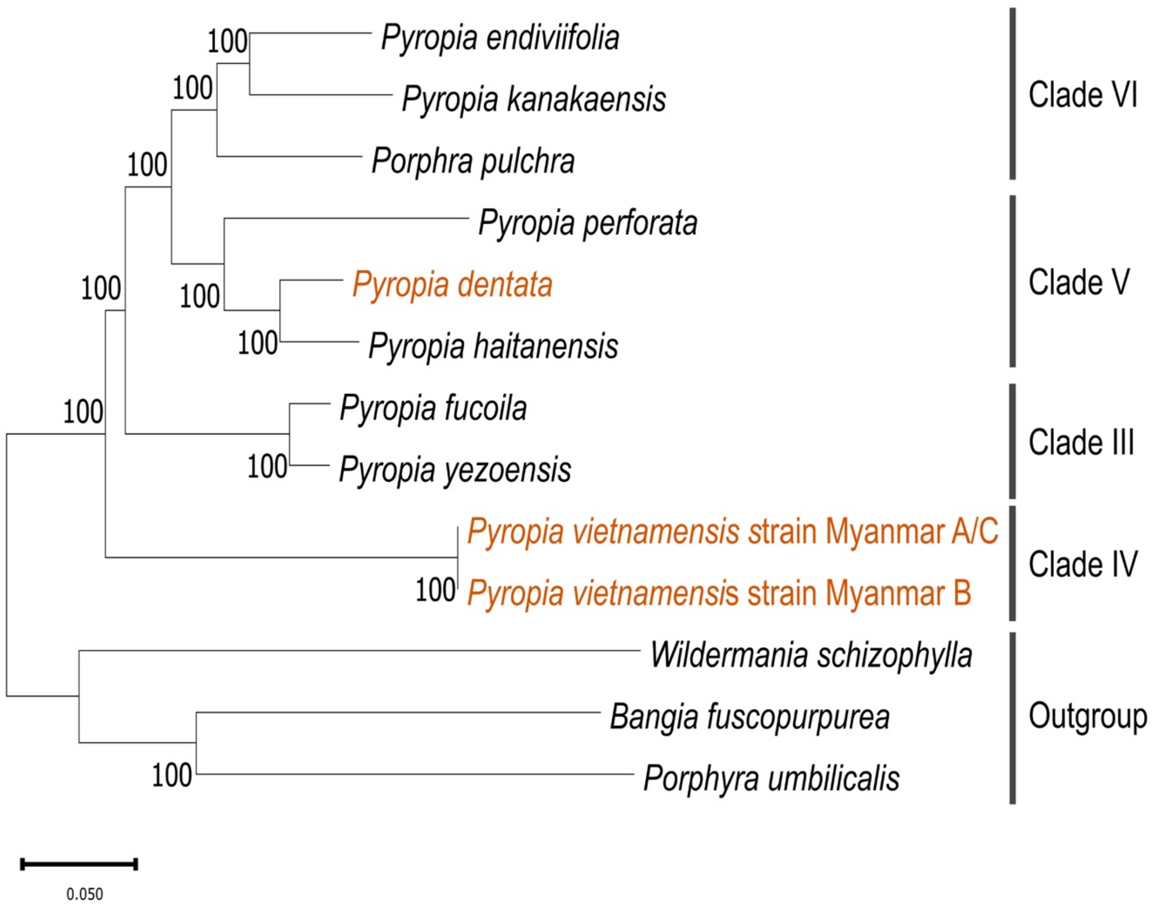

Supplementary Fig. S19. Phylogenetic tree of the mitochondrial genome of *Pyropia* species. An ML tree was constructed using the mitochondrial genome dataset of Myanmar seaweed and available sequence data on the database. ML bootstrap support values are presented at each node. The clade names used in the figure are those described by Yang et al.[1]. A total of 23,066 nucleotide positions (7,007 parsimony informative sites) were used in this analysis. The origin of the samples used in this analysis is described in Supplementary Table 6. The alignment data is available in fasta format as Supplementary Dataset 10, and ML tree is available in newick format as Supplementary Dataset 11.

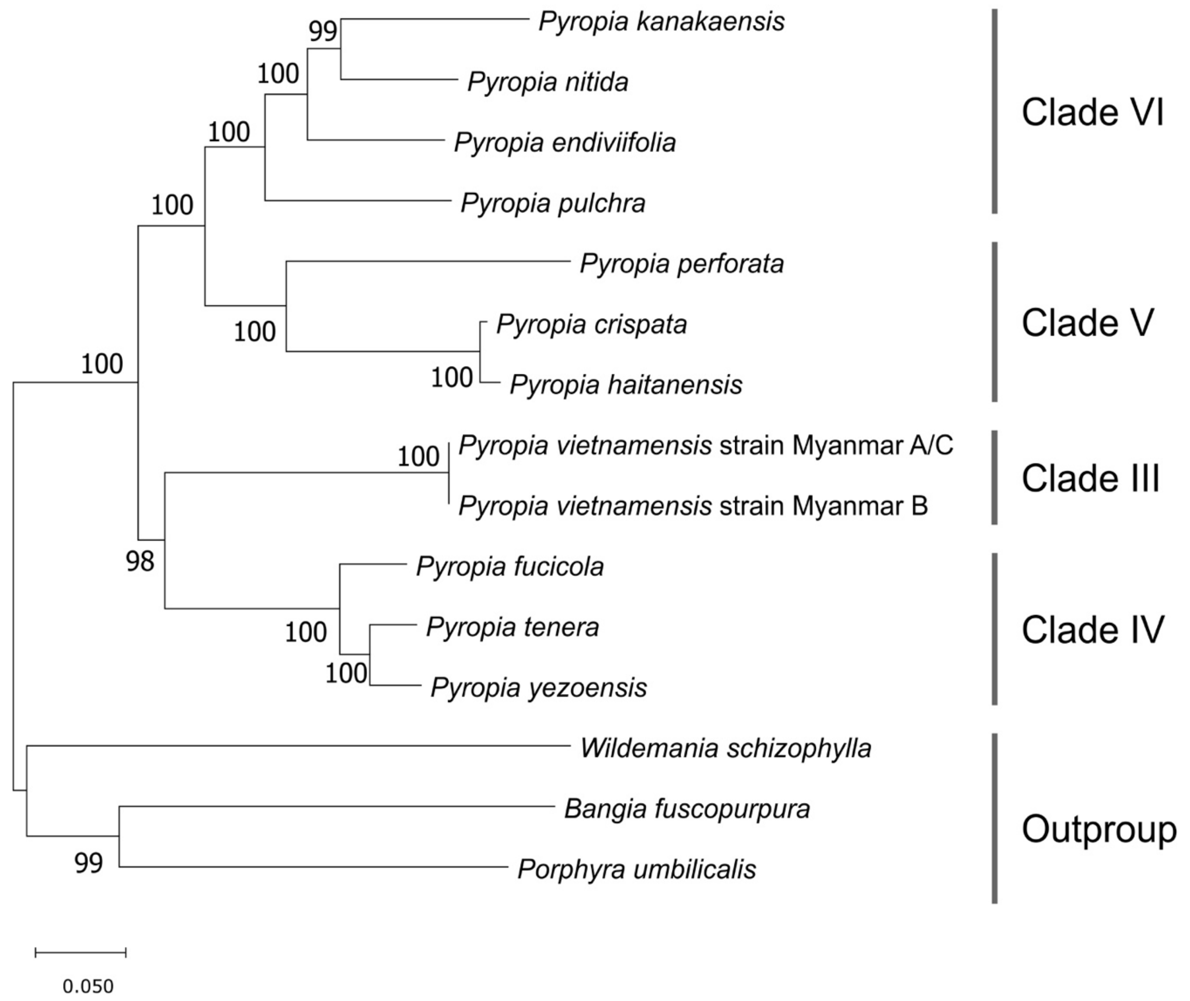

Supplementary Fig. S20. Comparison of topology among phylogenetic trees (cladograms). We compared the topology of five phylogenetic tree based on *rbcL* and nrSSU sequences from Sutherland et al. (2011)[8] and Zuccarello et al. (2022)[12] (A), *rbcL* and 18S sequences from Yang et al.[1] (2020) (B), and ML tree based on combination of *rbcL* and nrSSU sequences (C), ML trees based on organeller genomes (protein sequences) (D). In all figures, Clade I refers to *Uedaea*, Clade II refers to *Porphyrella*, Clade III refers to *Neopyropia*, Clade IV refers to *Calidia*, Clade V refers to *Neoporphyra*, and Clade VI refers to *Pyropia* genera, all of which are newly proposed names.

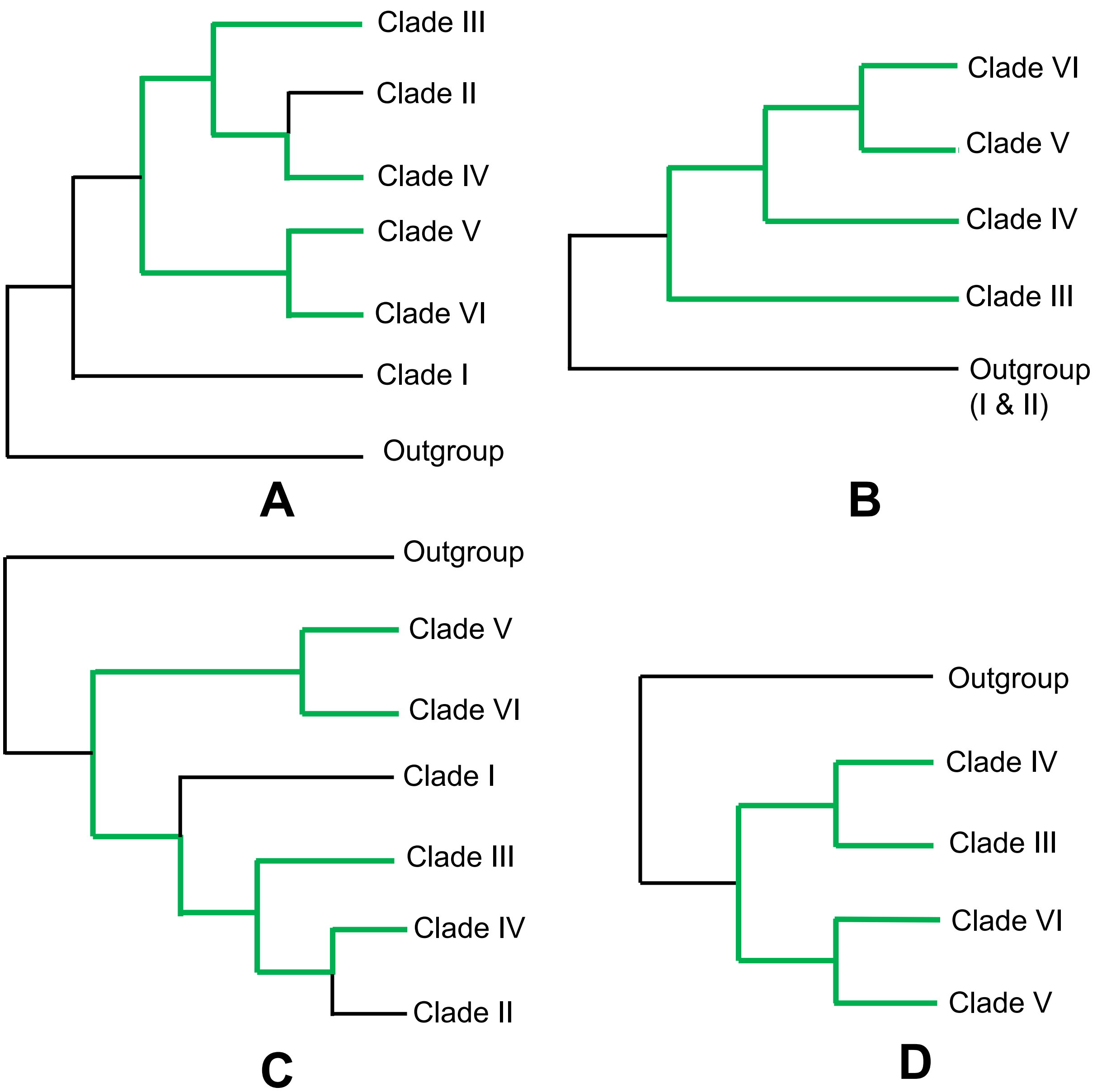

Supplement: Supplementary file 2 — Supplementary Information 2. [file 41598_2023_42262_MOESM2_ESM.pdf]
